# Metabolic changes in *Toxoplasma gondii*-infected host cells measured by autofluorescence imaging

**DOI:** 10.1128/mbio.00727-24

**Published:** 2024-07-08

**Authors:** Gina M. Gallego-López, Emmanuel Contreras Guzman, Danielle E. Desa, Laura J. Knoll, Melissa C. Skala

**Affiliations:** 1 Morgridge Institute for Research, Madison, Wisconsin, USA; 2 Department of Medical Microbiology & Immunology, University of Wisconsin–Madison, Madison, Wisconsin, USA; 3 Department of Biomedical Engineering, University of Wisconsin–Madison, Madison, Wisconsin, USA; University of Arizona, Tucson, Arizona, USA

**Keywords:** *Toxoplasma gondii*, host cell metabolism, redox biology, optical metabolic imaging (OMI), NAD(P)H-binding enzymes, reactive oxygen species

## Abstract

*Toxoplasma gondii,* the causative agent of toxoplasmosis, is an obligate intracellular parasite that infects warm-blooded vertebrates across the world. In humans, seropositivity rates of *T. gondii* range from 10% to 90% across communities. Despite its prevalence, few studies address how *T. gondii* infection changes the metabolism of host cells. In this study, we investigate how *T. gondii* manipulates the host cell metabolic environment by monitoring the metabolic response over time using noninvasive autofluorescence lifetime imaging of single cells, metabolite analysis, extracellular flux analysis, and reactive oxygen species (ROS) production. Autofluorescence lifetime imaging indicates that infected host cells become more oxidized and have an increased proportion of bound NAD(P)H compared to uninfected controls. Over time, infected cells also show decreases in levels of intracellular glucose and lactate, increases in oxygen consumption, and variability in ROS production. We further examined changes associated with the pre-invasion “kiss and spit” process using autofluorescence lifetime imaging, which also showed a more oxidized host cell with an increased proportion of bound NAD(P)H over 48 hours compared to uninfected controls, suggesting that metabolic changes in host cells are induced by *T. gondii* kiss and spit even without invasion.

**IMPORTANCE:**

This study sheds light on previously unexplored changes in host cell metabolism induced by *T. gondii* infection using noninvasive, label-free autofluorescence imaging. In this study, we use optical metabolic imaging (OMI) to measure the optical redox ratio (ORR) in conjunction with fluorescence lifetime imaging microscopy (FLIM) to noninvasively monitor single host cell response to *T. gondii* infection over 48 hours. Collectively, our results affirm the value of using autofluorescence lifetime imaging to noninvasively monitor metabolic changes in host cells over the time course of a microbial infection. Understanding this metabolic relationship between the host cell and the parasite could uncover new treatment and prevention options for *T. gondii* infections worldwide.

## INTRODUCTION

Host cells have evolved elaborate systems to counteract pathogen invasion, establishment, and replication, including phagolysosomal fusion, reactive oxygen species (ROS) production, nitrogen intermediates, sequestration of nutrients, and apoptosis (1–3). However, the host cell metabolic response to microorganism infection remains largely unclear, in part because it is challenging to noninvasively monitor host and parasite metabolism, especially in a live cell. In this study, we examine as an important case study how *Toxoplasma gondii* infection changes host cell metabolism, including redox balance and the binding activities of reduced nicotinamide adenine dinucleotide (NADH) and nicotinamide adenine dinucleotide phosphate (NADPH), collectively referred to as NAD(P)H, and flavin adenine dinucleotide (FAD). To do so, we harness the power of autofluorescence lifetime imaging of single cells over the time course of an infection, combined with the specificity of metabolite analysis, extracellular flux analysis, and ROS production.

*T. gondii*, the causative agent of toxoplasmosis, is an obligate intracellular parasite that infects warm-blooded vertebrates and establishes a silent, lifelong chronic infection in healthy humans. *T. gondii* has a global distribution, and its seropositivity rates range from less than 10% to over 90% depending on the location (4). The serum prevalence of *T. gondii* depends on a variety of factors such as eating habits, the presence of the definitive host (felines), and environmental conditions (5). Infection acquired during pregnancy can be particularly harmful because the parasite can cross the placental barrier and infect the fetus, causing retinal inflammation, encephalitis, mental and physical disabilities, and potentially death (6). Another population at high risk is immunocompromised individuals, in whom the parasite cyst stage can reactivate and cause neurologic defects and seizures (7). As an obligate intracellular parasite, *T. gondii* relies on the host cell to provide many metabolites such as arginine, tyrosine, tryptophan, purines, cholesterol, or sphingolipids (8–13). To scavenge these metabolites, the parasite causes a remodeling of the infected host cell. Therefore, understanding this metabolic relationship between the host cell and the parasite could uncover new treatment and prevention options for *T. gondii* infections worldwide.

Though the redox biology of the host response to *T. gondii* infection is underexplored, the association of the mitochondria in *T. gondii* and overall host reprogramming upon infection has been characterized previously. The former involves *T. gondii* mitochondrial association factor (MAF) and 13 or more host proteins (14–17). In the RH *T. gondii* strain, used here as a control, host mitochondria relocalize around the parasite-containing vacuoles upon infection (16). Though the cause and purpose of mitochondria elongation is not fully clear, it could be the parasite’s strategy to induce host lipophagy; acquire fatty acids, amino acids, or pyruvate; or alternatively a host defense mechanism that induces host mitochondria fusion, which limits parasite proliferation (18). The ME49 *T. gondii* strain investigated here does not have this phenotype or the MAF genes (17). Blader *et al*. have classified host genes modulated in response to *T. gondii* infection into three functionally different classes: (i) genes required for host defense; (ii) genes required for parasite growth; and (iii) genes incidentally regulated as a consequence of modulating the first two classes (19). Quantitative proteomic studies have suggested a global reprogramming of the cell metabolism by the parasite (1), and we recently showed how a *T. gondii* full infection (20) and the pre-invasion “kiss and spit” process (21) produces significant changes in host metabolites.

However, the host metabolic changes that *T. gondii* infection induces over time have not yet been examined by live single-cell imaging. In this study, we use optical metabolic imaging (OMI) to measure the optical redox ratio (ORR) in conjunction with fluorescence lifetime imaging microscopy (FLIM) of NAD(P)H and FAD to noninvasively monitor single host cell response to *T. gondii* infection over 48 hours. The ORR is defined as the fluorescence intensity of NAD(P)H / (FAD +NAD(P)H) (Table 1) (22–25). NADH and NADPH are spectrally indistinguishable and are collectively referred to as NAD(P)H (26, 27). Many factors can change the ORR, such as hypoxia, high carbon demands, increased proliferation rate, and fatty acid synthesis (24). As an indicator of the oxidation–reduction state of the cell and an important marker of cell health that can be used to monitor living tissues and cells, the ORR has been used to study numerous biological processes including cancer, thermal stress, *de novo* fatty acid synthesis, and diabetes (22–24, 28). ORR imaging has also been previously used in infectious disease research to monitor oxidative stress in host cells with chronic infection with hepatitis C virus (HCV) (29). FLIM is sensitive to the protein-binding activities of NAD(P)H and FAD (30, 31). Specifically, NAD(P)H has a short lifetime in the free conformation (τ_1_) and a long lifetime in the protein-bound conformation (τ_2_), while the converse is true for FAD (τ_1_ is bound, and τ_2_ is free). Due to these distinct lifetimes, FLIM can quantify the relative fractions of free and protein-bound NAD(P)H and FAD in the cell (Table 1), providing a snapshot of enzyme binding activity (32). FLIM has been previously used in infectious disease studies of HCV (33), *Chlamydia trachomatis* infection (34), and *Plasmodium falciparum* replication (35).

**TABLE 1:** OMI parameters and definitions

Parameter	Description	
	NAD(P)H	FAD
τ_1_	Free lifetime	Protein-bound lifetime
τ_2_	Protein-bound lifetime	Free lifetime
α_1_	Proportion of free NAD(P)H	Proportion of bound FAD
α_2_	Proportion of bound NAD(P)H	Proportion of free FAD
I	Absolute intensity	Absolute intensity
τ_m_	Mean lifetime = α_1_ τ_1_ + α_2_ τ_2_	
Optical redox ratio	ORR = I_NAD(P)H_ / (I_FAD_ +I_NAD(P)H_)	

This study seeks to answer the following research questions: (1) Does *T. gondii* infection affect the redox balance of the host cell? (2) Does *T. gondii* infection affect the NAD(P)H or FAD binding activities of the host cell? (3) Does *T. gondii* infection induce changes over time in host cell glucose consumption, lactate secretion, oxygen consumption, and/or ROS production? To answer these questions, we imaged ME49 *T. gondii*-infected human foreskin fibroblast (HFF) cells with OMI over 48 hours. Comparisons of *T. gondii* full infection and the pre-invasion “kiss and spit” process were similarly performed with OMI. Additional measurements of intracellular and extracellular glucose and lactate, extracellular flux analysis, and ROS production support live cell imaging results. This research demonstrates the significance of monitoring metabolic changes induced during *T. gondii* infection in live cells.

## RESULTS

### Image analysis to quantify intracellular *T. gondii*

First, we established *T. gondii* infection in quiescent HFF cells using ME49 mCherry-labeled parasites because mCherry fluorescence is spectrally separate from both NAD(P)H and FAD fluorescence (36). FAD and NAD(P)H intensities and lifetimes of infected cells were obtained using two-photon FLIM. OMI was collected at 1, 6, 9, 12, 24, and 48 hours post-infection (HPI) in two independent experiments. We generated whole cell masks using the NAD(P)H intensity images and *T. gondii* masks based on the mCherry signal (Fig. S1). The masks were used to quantify *T. gondii* at the single-cell level.

### Establishing a *T. gondii* infection threshold

Second, we defined the percentage of the intracellular *T. gondii* area by cell area (Fig. S2A). Variations in the percentage of the intracellular parasite showed that *T. gondii* did not infect quiescent HFF cells equally. To compare the infection distribution across the cells, we plotted histograms of percentage of intracellular *T. gondii* in each individual cell for each timepoint for each experiment (Fig. S2B, S3 to S5).

Given that not all quiescent HFF cells were equally infected by the parasite at the same timepoint, we grouped cells into low- and high-infection categories by setting a threshold on the percentage of intracellular *T. gondii*. In choosing a threshold, we accounted for a low signal-to-noise ratio in mCherry-labeled *T. gondii* images, which led to erroneous quantification of some background red fluorescence pixels as the parasite. To prevent false positives for infected cells, we compared OMI parameters (ORR, NAD(P)H, and FAD τ_m,_ τ_1,_ τ_2,_ α_1,_ and α_2_) between cells with 5% mCherry pixels and 10% mCherry pixels but did not observe any significant differences between these thresholds (data not shown). We selected 5% as our cutoff threshold because of the similar OMI parameter results and to increase the likelihood of including true intracellular parasites. We used this analysis to empirically determine that those cells with lower than 5% mCherry pixels had no significant *T. gondii* infection or that the infection could be a false-positive, as above.

### OMI changes in low vs high *T. gondii-*infected cells

**Fig 1:**
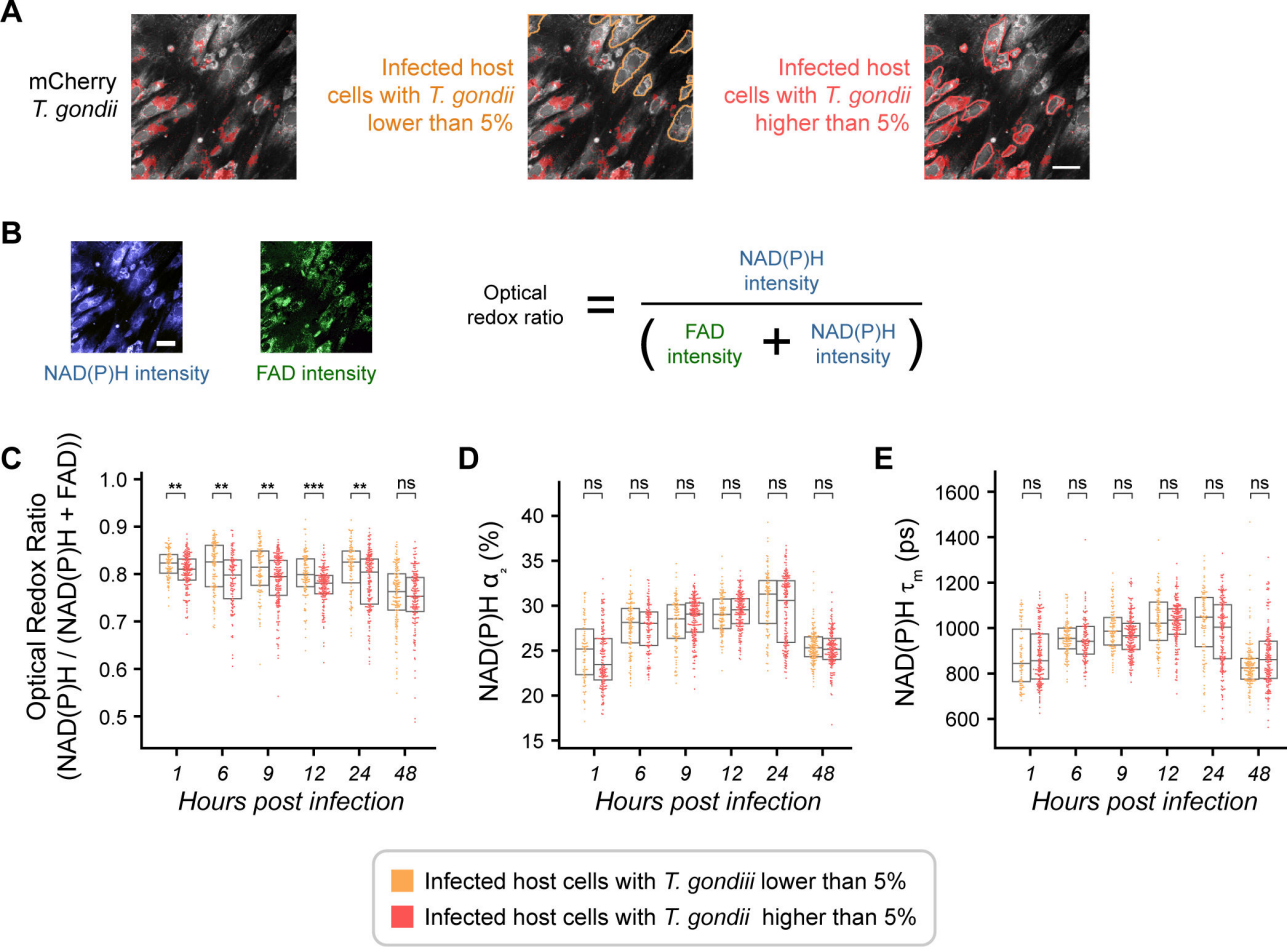
Establishing a threshold of *Toxoplasma gondii* infection. (**A**) Representative images of infected HFF cells with lower than 5% (yellow) and higher than 5% (red) mCherry *T. gondii*. The same image from reference 104 has been used for all conditions for ease of comparison and to illustrate the analysis workflow. Scale bar = 50 µm. (**B**) ORR [fluorescence intensity of NAD(P)H/(FAD +NAD(P)H)]. (**C**) ORR of *T. gondii*-infected HFF cells with low vs high intracellular *Toxoplasma gondii* over the time course of infection. (**D**) Percentage of protein-bound NAD(P)H (α_2_) of *T. gondii*-infected HFF cells with low vs high *Toxoplasma gondii* over the time course of infection. (**E**) Mean lifetime of NAD(P)H (τ_m_) of *T. gondii*-infected HFF cells with lower than 5% (yellow) vs higher than 5% (red) intracellular *T. gondii* over the time course of infection. Each dot represents a host cell. Error bars represent the median and 95% confidence. Statistical significance was determined by Student’s *t*-test adjusted for multiple comparisons with a Bonferroni correction. ns, *P ≤* 1.00e + 00; *, 1.00e-02 < *P ≤* 5.00e-02; **, 1.00e-03 *< P ≤* 1.00e-02; ***, 1.00e-04 *< P ≤* 1.00e-03; ****, *P ≤* 1.00e-04*.* Cell count low *T. gondii* = 632; cell count high *T. gondii* = 974.

Having categorized cells in the infected condition, we then compared low vs.high parasite-infected cells. OMI parameters were calculated as detailed in Fig. S6. Figure 1A shows representative images outlining cells with low vs high infection with a 5% threshold. Multiple definitions of the ORR exist, but in this study, we used NAD(P)H/(NAD(P)H + FAD) (Table 1; Fig. 1B) (37). The ORR is an intensity measurement, so comparing to a daily control accounts for system drift (38). Within a timepoint, cells with high infection showed greater oxidation (i.e., decreased ORR) compared to cells with low infection at 1 through 24 hours post-infection (Fig. 1C). We did not find significant differences for NAD(P)H mean lifetime (τ_m_) or the proportion of protein-bound NAD(P)H (α_2_) (Table 1) between low and high infection (Fig. 1D and E).

### Whole-cell and host cell analyses show similar changes with *T. gondii* infection

We used two approaches to analyze changes in OMI parameters in infected host cells. First, we analyzed the host plus parasite metabolism in the infected cells, without distinguishing between host or intracellular parasite pixels, which we refer to as whole-cell analysis (Fig. S7A through D). Second, to distinguish metabolic changes present only in the host, we obtained a new host cell mask by multiplying the whole cell mask by an inverted *T. gondii* mask, which we refer to as host cell analysis (Fig. S7A, E through G). In the host cell analysis, we subtract the intracellular *T. gondii* pixels that represent an intracellular parasite or a group of intracellular parasites multiplying within vacuoles. Given this distinction, we were surprised to find that both approaches resulted in similar trends of increased oxidative metabolism.

We believe that the reason underlying the similar results for both whole cell and host cell analyses is that the parasite induced metabolic changes in the host cell even without invasion, as has been documented previously (21, 39), and these changes go beyond the parasite pixels. That said, we continue using whole-cell analysis for this study because it also allows us to compare our results with those of other techniques that do not separate the intracellular microorganism from the host cell.

### *T. gondii* infection changes the redox balance of the host cell

**Fig 2:**
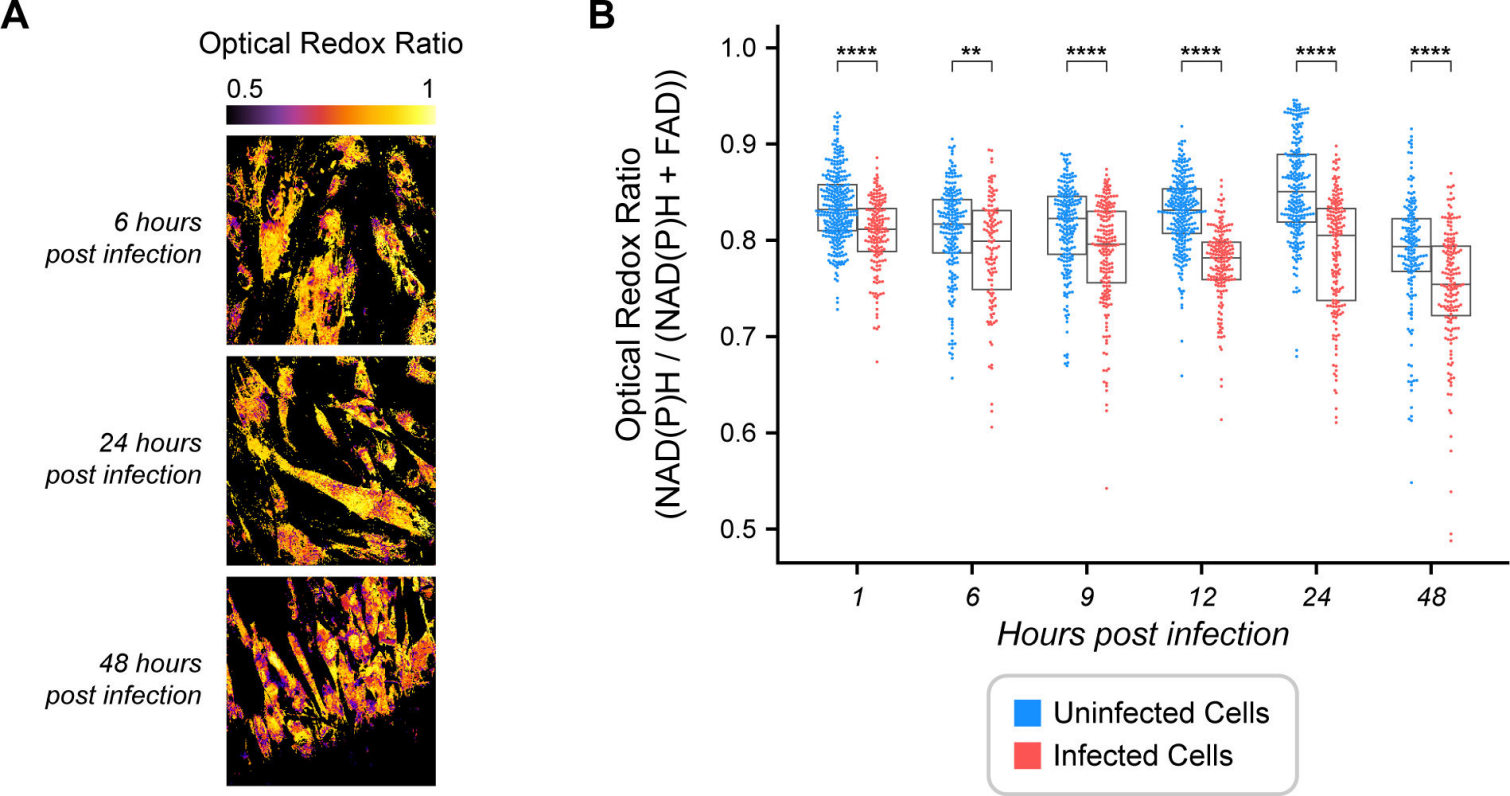
Optical redox ratio (ORR) temporal changes in *Toxoplasma gondii-*infected cells. (**A**) Representative images of temporal changes in the ORR of *T. gondii-*infected HFF cells. A lower ORR means a more oxidized environment, and a higher ORR means a more reduced environment. Scale bar = 50 µm. (**B**) ORR of *T. gondii*-infected HFF cells vs uninfected cells over a time course of infection by the whole-cell approach. Uninfected HFF cells are represented in blue, and infected HFF cells are represented in red. Each dot represents a host cell. Total number of cells *n* = 243, 225, 285, 277, 263, and 312 for 1, 6, 9, 12, 24, and 48 HPI, respectively. These results represent two independent experiments. Error bars represent the median and 95% confidence. Statistical significance was determined by Student’s *t*-test adjusted for multiple comparisons with a Bonferroni correction. ns, *P ≤* 1.00e + 00; *, 1.00e-02 *< P ≤* 5.00e-02; **, 1.00e-03 *< P ≤* 1.00e-02; ***, 1.00e-04 *< P ≤* 1.00e-03; ****, *P ≤* 1.00e-04.

Using whole-cell analysis and our previously established intracellular parasite threshold of 5% to differentiate cells with low vs high infection, we next compared uninfected HFF cells with high ME49 *T. gondii-*infected cells (Fig. 2A). Single-cell analysis found that infected cells are more oxidized compared to uninfected cells within each timepoint at 1 through 48 HPI (Fig. 2B). This intracellular oxidation in high-infected cells compared to uninfected controls is consistent with the oxidation of high infection compared to low infection (Fig. 1C).

### NAD(P)H mean lifetime and the proportion of protein-bound NAD(P)H increase in *T. gondii*-infected HFF cells

**Fig 3:**
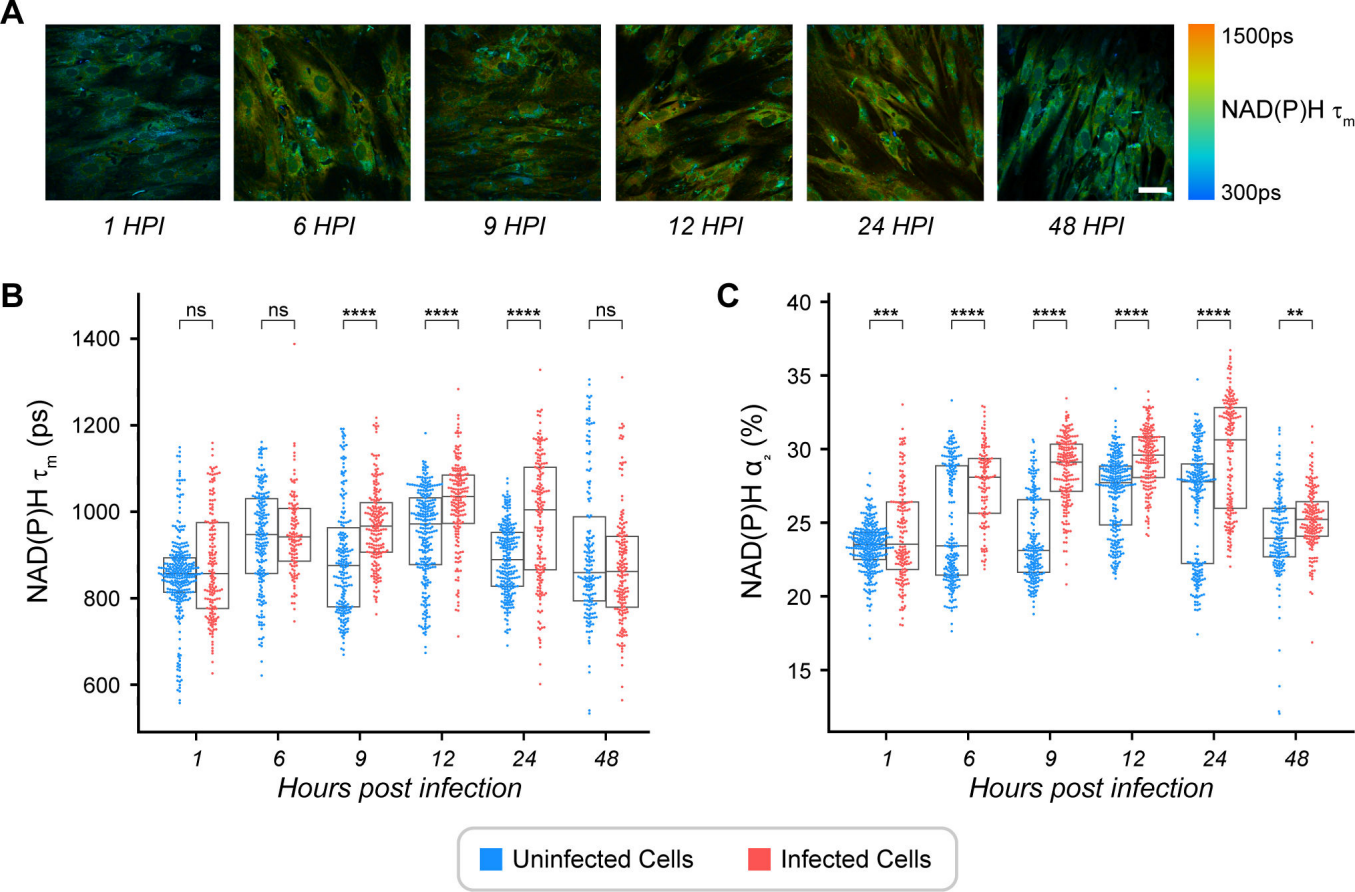
Temporal changes in NAD(P)H lifetime of *Toxoplasma gondii*-infected HFF cells. (**A**) Representative images of NAD(P)H mean lifetime (τ_m_) reported in picoseconds (ps) for 1 to 48 hours post-infection (HPI). (**B**) NAD(P)H mean lifetime (τ_m_) of *T. gondii*-infected HFF cells in a 48-HPI time course experiment by the whole-cell approach. (**C**) Percentage of protein-bound NAD(P)H (α_2_) of *T. gondii*-infected HFF cells in a 48-HPI time course experiment by the whole cell approach. Uninfected HFF cells are represented in blue, and infected HFF cells (with *T. gondii* higher than 5%) are represented in red. Each dot represents a host cell. Total number of cells *n* = 243, 225, 285, 277, 263, and 312 for 1, 6, 9, 12, 24, and 48 HPI, respectively. These results represent two independent experiments. Scale bar = 50 µm. Error bars represent the median and 95% confidence. Statistical significance was determined by Student’s *t*-test adjusted for multiple comparisons with a Bonferroni correction. ns, *P ≤* 1.00e + 00; *, 1.00e-02 *< P ≤* 5.00e-02; **, 1.00e-03 *< P ≤* 1.00e-02; ***, 1.00e-04 *< P ≤* 1.00e-03; ****, *P ≤* 1.00e-04*.*

We obtained FLIM images (Fig. 3A) and performed single-cell analyses of NAD(P)H τ_m_ (Fig. 3B) in high ME49 *T. gondii*-infected cells and uninfected HFF cells. Within a timepoint, NAD(P)H τ_m_ increased significantly in infected HFF cells compared to uninfected HFF cells at 9, 12, and 24 HPI (Fig. 3B). NAD(P)H α_2_ in infected HFF cells was greater than that in uninfected HFF cells at all timepoints (Fig. 3C). The more oxidized ORR along with increases in NAD(P)H τ_m_ and NAD(P)H α_2_ all indicate increases in mitochondrial metabolism with infection (22, 40). Changes in FAD fluorescence lifetimes in infected cells compared to uninfected cells vary between 1 and 48 HPI, which reflects differences in the use of FAD during infection (Fig. S8).

### *T. gondii* kiss and spit modifies the ORR and NAD(P)H lifetime of the host cell

**Fig 4:**
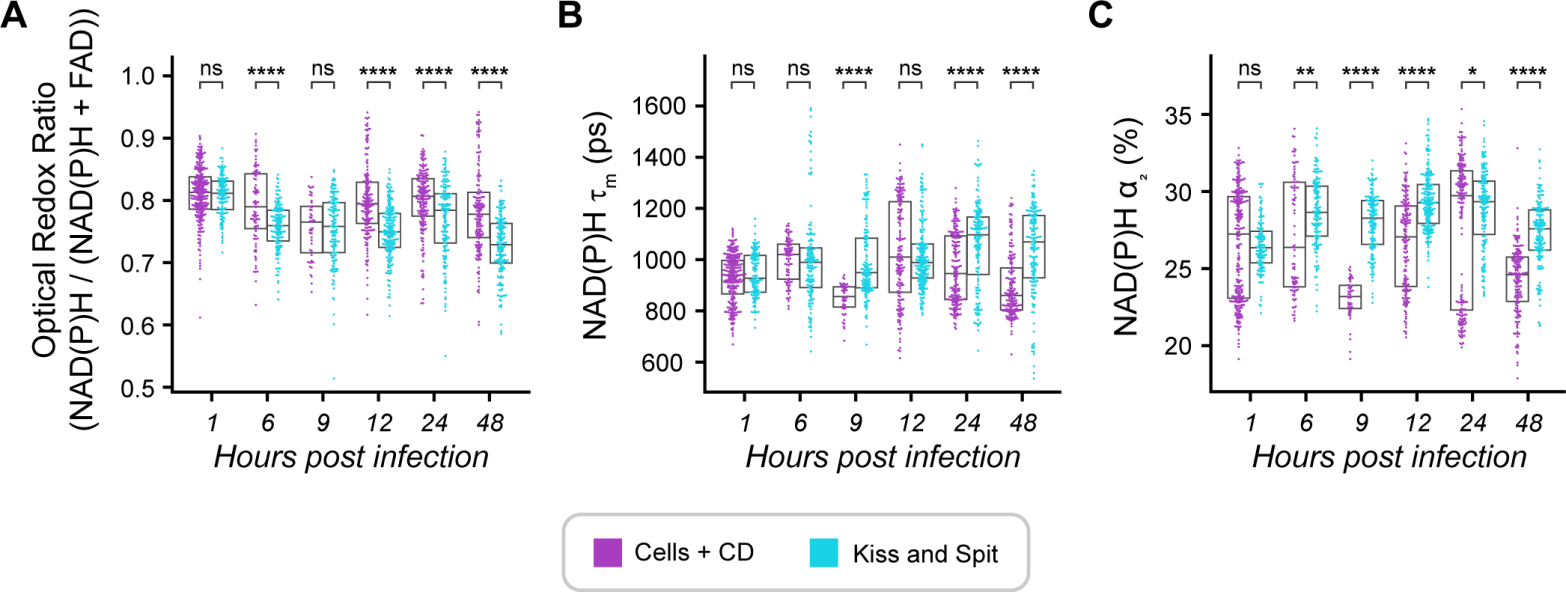
Temporal changes in OMI parameters during *Toxoplasma gondii* kiss and spit. (**A**) Comparison of temporal ORR changes in kiss and spit host cells and in control of kiss and spit (Cell +cytochalasin D (CD)). (**B**) Temporal changes in mean lifetime of NAD(P)H (τ_m_) during *T. gondii* kiss and spit. (**C**) Temporal changes in the percentage of protein-bound NAD(P)H (α_2_) during *T. gondii* kiss and spit. HFF cells treated with the inhibitor cytochalasin D (CD) are represented in purple, and HFF cells with active *T. gondii* kiss and spit are represented in cyan. These results represent two independent experiments. Each dot represents a host cell. Error bars represent the median and 95% confidence. Statistical significance was determined by Student’s *t*-test adjusted for multiple comparisons with a Bonferroni correction. ns, *P ≤* 1.00e + 00; *, 1.00e-02 *< P ≤* 5.00e-02; **, 1.00e-03 *< P ≤* 1.00e-02; ***, 1.00e-04 *< P ≤* 1.00e-03; ****, *P ≤* 1.00e-04.

We performed an additional analysis “kiss and spit” using an actin polymerization inhibitor, cytochalasin D, which allows *T. gondii* to secrete the contents of their rhoptries into host cells while preventing infection (41). Like full infection, we found that kiss and spit created a more oxidized intracellular environment compared to cells treated with cytochalasin D at 6, 12, 24, and 48 HPI (Fig. 4A; Fig. S9A). Additionally, kiss and spit increased NAD(P)H τ_m_ at 9, 24, and 48 HPI (Fig. 4B; Fig. S9B) and NAD(P)H α_2_ at 6, 9, 12, 24, and 48 HPI (Fig. 4C; Fig. S9C) compared to cells treated with cytochalasin D. Overall, these changes in the ORR and NAD(P)H lifetimes with kiss and spit (Fig. 4) are consistent with changes observed in full infection (Fig. 2 and 3 ).

### *T. gondii* infection alters host glucose consumption and lactate production

**Fig 5:**
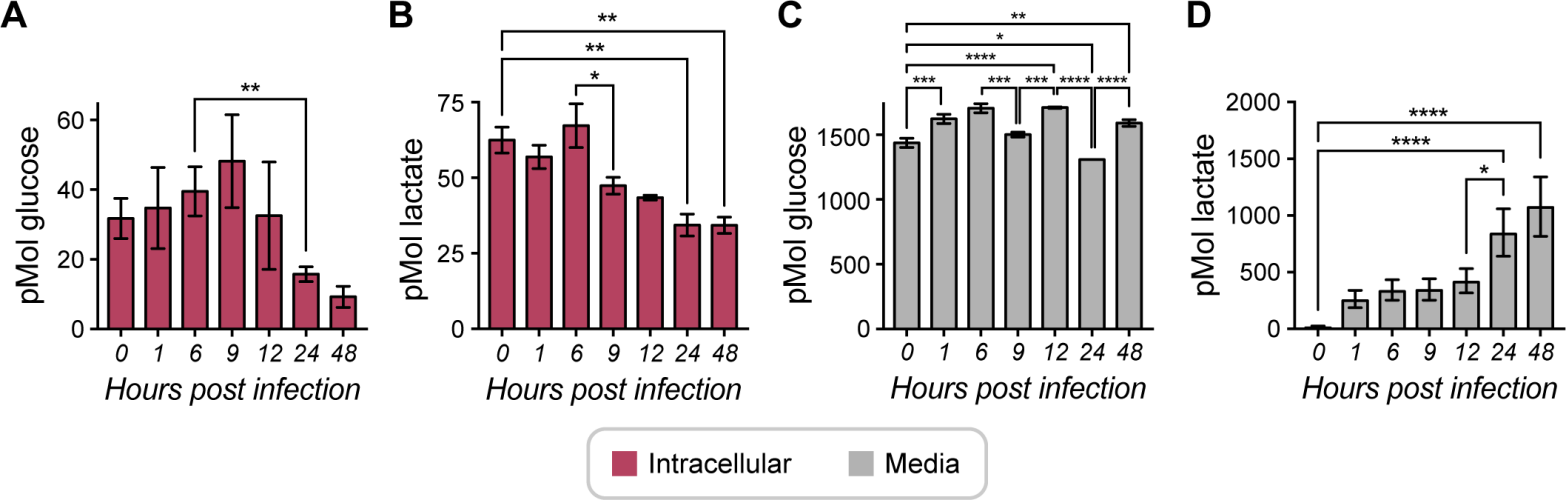
Glucose and lactate production in ME49 *Toxoplasma gondii-*infected host cells during the course of infection. (**A**) Intracellular production of glucose in ME49 *T. gondii-*infected HFF host cells during the time course of infection, *n* = 6–15 wells. (**B**) Intracellular production of lactate in ME49 *T. gondii-*infected HFF host cells during the time course of infection, *n* = 6 wells. (**C**) Glucose in media in ME49 *T. gondii-*infected HFF host cells during the time course of infection, *n* = 4 wells. (**D**) Lactate in extracellular media in ME49 *T. gondii-*infected HFF host cells during the time course of infection, *n* = 6 wells. Each bar represents the mean of n replicates, and error bars represent the SEM. Statistical analysis was performed by one-way ANOVA, and multiple comparisons were performed by Tukey’s test. 0.1234 (ns), 0.0332 (*), 0.0021 (**), 0.0002 (***), and <0.0001 (****).

Given that OMI revealed changes in host cell metabolism with infection at all timepoints (Fig. 2 and 3 ), we next analyzed intracellular and extracellular glucose and lactate changes during infection (Fig. 5). Intracellular glucose concentrations revealed a slight increase until 9 HPI and then dropped significantly in ME49 *T. gondii*-infected cells (Fig. 5A). Intracellular lactate concentrations decreased after 6 HPI (*P* = 0.0553) in ME49 *T. gondii*-infected cells (Fig. 5B). While the extracellular glucose concentration varies slightly during the 48-hour time course infection (Fig. 5C), the concentration of extracellular lactate increased significantly after 12 hours of infection (Fig. 5D). It is possible that the increases in glucose in media (Fig. 5C) are related to gluconeogenesis because *T. gondii* tachyzoites can assimilate carbon in the absence of glycolysis by glutaminolysis-derived gluconeogenesis (42–44) and lactate-derived gluconeogenesis (Fig. 5D) (45, 46). Additionally, the increase in both glucose and lactate in media at 48 HPI could be related to host cell lysis and the presence of extracellular parasites.

### *T. gondii* infection alters host mitochondrial and glycolytic activity

We next analyzed mitochondrial and glycolytic functions to understand how *T. gondii* infection changes host cell metabolism over time. Previous studies have demonstrated that changes in ORR and NAD(P)H lifetimes correlate with changes in mitochondrial respiration and glycolysis, as measured by extracellular flux analysis (22, 47–49). These measurements have been made before with extracellular *T. gondii* (50), but not in infected cells or in intracellular parasites. We used a Seahorse XFp extracellular flux analyzer to measure the mitochondrial and glycolytic functions of quiescent HFF host cells infected with the wild-type ME49 *T. gondii* strain. As a control, we used a type-I RH *T. gondii* strain without and with a deletion in mitochondrial association factor 1 (RHΔMAF) (17). These experiments complement intracellular and extracellular glucose and lactate measurements.

**Fig 6:**
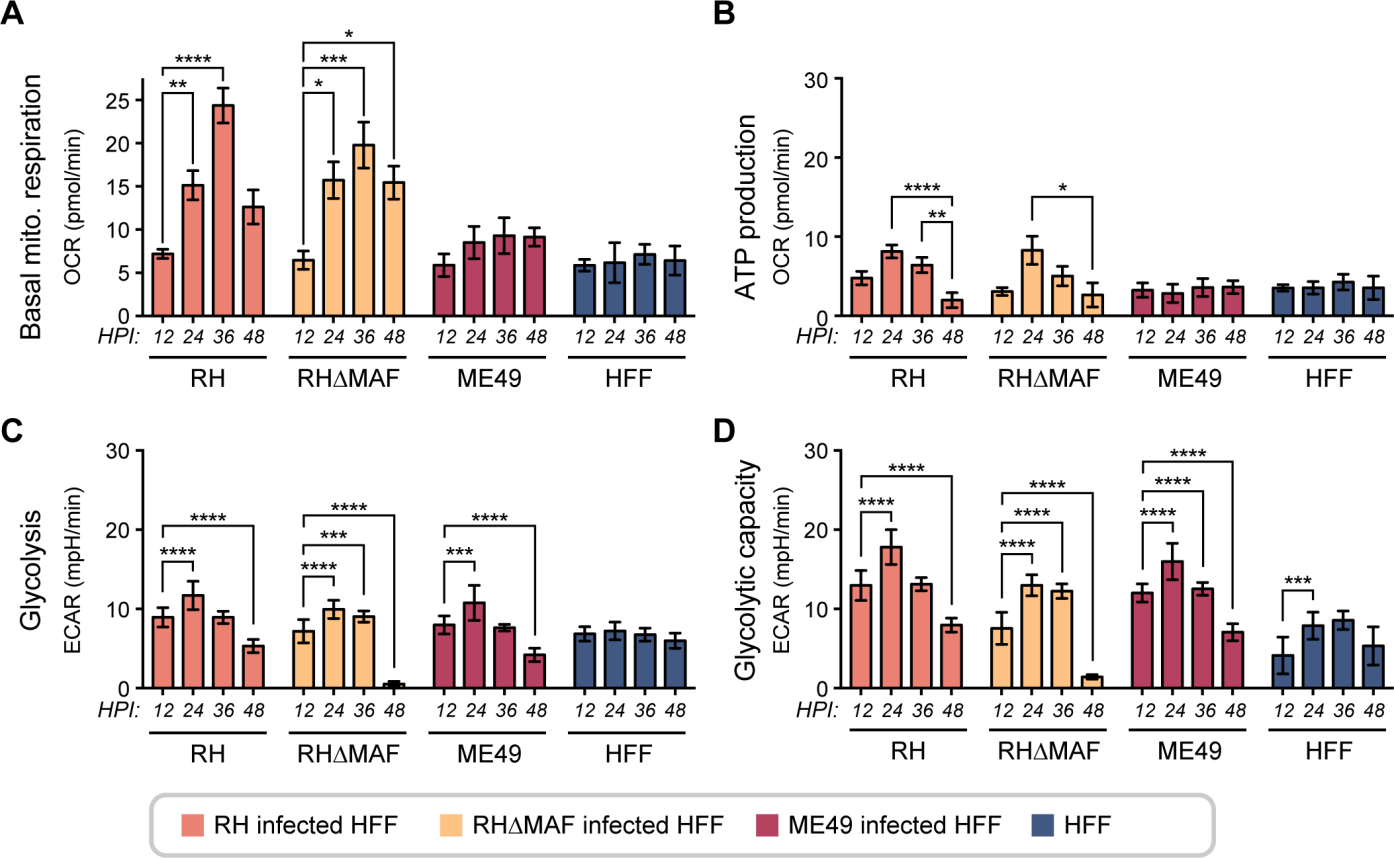
Temporal mitochondrial and glycolytic changes in infected HFF cells by different strains of *Toxoplasma gondii* during the time course of infection. (**A**) Basal oxygen consumption rate. (**B**) Mitochondrial ATP production. (**C**) Glycolysis. (**D**) Glycolytic capacity. HFF cells were infected with one of three *T. gondii* strains: ME49, RHΔMAF, or RH. The oxygen consumption rate (OCR) was calculated using a Seahorse Mito Stress Test, and the extracellular acidification rate (ECAR) was calculated using a Seahorse Glycolysis Stress Test. Each bar represents the mean of 12 replicates, and error bars represent the SEM. Statistical analysis was performed by ANOVA with Tukey’s test to compare timepoints in each strain. 0.1234 (ns), 0.0332 (*), 0.0021 (**), 0.0002 (***), and <0.0001 (****).

We performed a mitochondrial stress test at multiple timepoints (Fig. 6) and observed that RH and RHΔMAF *T. gondii*-infected HFF cells showed progressive increases in basal mitochondrial respiration levels until 36 HPI and then a reduction at 48 HPI (Fig. 6A). ME49 *T. gondii*-infected HFF cells showed low basal mitochondrial respiration level and did not fluctuate over time, which indicates lower mitochondrial energetic demand in the first 48 HPI (Fig. 6A). It is also possible that the resolution of this extracellular flux technique does not capture small changes in mitochondrial respiration in this strain. Overall, these data suggest more mitochondrial activity in RH *T. gondii*-infected cells than in ME49 *T. gondii*-infected cells in the first 48 HPI. Uninfected quiescent HFF cells showed low mitochondrial respiration as expected, suggesting that all changes observed correspond to the effect of intracellular parasites on host cells.

The mitochondrial stress test also calculated mitochondrial ATP production in infected cells over time (Fig. 6B). RH and RHΔMAF *T. gondii-*infected HFF cells showed significant fluctuations in mitochondrial ATP over 48 HPI (Fig. 6B). Mitochondrial ATP production in ME49 *T. gondii*-infected HFF cells did not fluctuate over time, like the uninfected cells (Fig. 6B). Additional metabolic parameters evaluated included the non-mitochondrial oxygen consumption (Fig. S10A), which is calculated when the respiratory chain is totally inhibited with rotenone and antimycin A, and proton leak, which is associated with mitochondrial damage and pathogenesis (51–53) (Fig. S10B). Both measures showed significant changes over time in HFF cells infected with both RH *T. gondii* strains, but not the ME49 *T. gondii* strain.

To evaluate glycolytic activity, we measured the extracellular acidification rate (ECAR) with a glycolysis stress kit at multiple timepoints (Fig. 6C and D; Fig. S10C). First, we measured glycolysis, which is the ECAR reached by a given cell after the addition of saturating amounts of glucose (54–57). Uninfected cells did not show changes in glycolysis, but similar fluctuations were observed among cells infected with each of the three *T. gondii* strains (Fig. 6C). The significant decrease in glycolysis between 12 and 48 HPI in ME49 *T. gondii*-infected cells (Fig. 6C, *P* < 0.0001) aligns with the decrease in intracellular glucose (Fig. 5A). Second, we measured glycolytic capacity, which is the maximum ECAR reached by a cell shutting down oxidative phosphorylation and driving the cell to use glycolysis to its maximum capacity (54–57). We found significant changes in glycolytic capacity of all *T. gondii*-infected HFF cells over time (Fig. 6D). Third, we measured glycolytic reserve (Fig. S10C) or the capability to respond to an energy demand such as the presence of a intracellular parasite like *T. gondii* (55–57). Glycolytic reserve increased at 48 HPI for RH and ME49 but decreased at 48 HPI for RHΔMAF *T. gondii*-infected cells (Fig. S10C). In summary, we observed that *T. gondii* infection induces drastic host changes in glycolysis, glycolytic capacity, and glycolytic reserve during the first 48 hours of infection.

Note that the decrease in mitochondrial respiration and glycolysis from 36 to 48 HPI in RH and RHΔMAF *T. gondii*-infected cells could be related to egress of parasites and cell death. RH and ME49 *T. gondii* strains have different kinetics, dynamics, growth rates, and duplicating times (58–62); for this reason, we cannot compare strains across metabolic flux experiments. We note that during the first 48 HPI, there are host changes at the level of mitochondrial respiration and glycolysis, which are distinct according to the strain of *T. gondii* (Fig. 6).

### *T. gondii* infection alters ROS in the host cell

**Fig 7:**
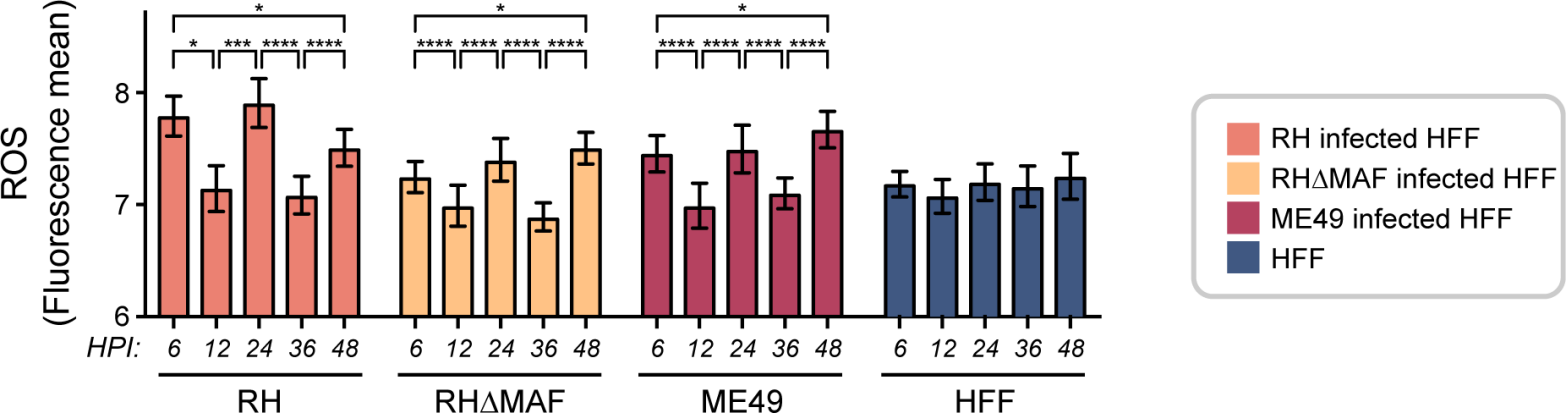
Reactive oxygen species (ROS) production in HFF infected with three different strains of *Toxoplasma gondii* during the time course of infection. CellRox green fluorescence intensity mean was measured. HFF cells were infected with ME49, RHΔMAF, or RH *T. gondii* strains, Uninfected quiescent HFF cells were evaluated as a negative control. Each bar represents the mean of four replicates, and error bars represent the SEM. Statistical analysis was performed by one-way ANOVA with Tukey’s multiple comparison test to compare timepoints in each strain. 0.1234 (ns), 0.0332 (*), 0.0021 (**), 0.0002 (***), and <0.0001 (****).

Because other cytosolic mechanisms also consume oxygen and affect the redox biology of the cell (63), we also measured ROS production in infected cells. To understand the contribution of host cell ROS production on the metabolic reprogramming of the host cell induced by *T. gondii* infection, including the oxidized ORR and increased NAD(P)H τ_m_ and α_2_, we measured intracellular ROS in ME49 *T. gondii*-infected HFF cells. We used a green fluorescence probe that predominantly detects hydroxyl radicals and superoxide anions, detects to a low extent of tert-butyl-hydroperoxide, and does not detect hydrogen peroxide. As a control, we used a *T. gondii* type-I RH strain with and without a deletion in mitochondrial association factor 1 (RHΔMAF) (17). ROS production fluctuated over time in HFF cells infected with the three *T. gondii* strains (Fig. 7), consistent with prior work (64–66).

### Gene expression analysis confirms the redox and NAD(P)H binding changes in ME49 *T. gondii-*infected cells

In order to better understand the molecular mechanisms behind the changes observed in the data previously, we compared the results of this study to our previously published RNA sequencing data on the same quiescent host cell system infected with ME49 *T. gondii* (20) and performed five new different analyses. First, using the Database for Annotation, Visualization, and Integrated Discovery (DAVID) (67), we analyzed the gene ontology (GO) terms related to molecular function of genes more abundant in ME49 *T. gondii*-infected HFF cells (Fig. S11). At all timepoints, we observed increased abundance of GO terms related to key aspects of oxidative phosphorylation, such as NADH dehydrogenases and hydrogen ion transmembrane transport. These NADH dehydrogenase enzymes could be binding to NADH, resulting in the increase in NAD(P)H α_2_ (Fig. 3C). Second, we analyzed the reactome of genes upregulated in ME49 *T. gondii*-infected HFF cells (Fig. S12). At all timepoints, we observed an increased abundance of genes clustered in distinct categories such as respiratory electron transport, TCA cycle, and cellular response to stress. These genes are upregulated in response to *T. gondii* infection and affect the redox balance of host cells (Fig. S12). Third, we analyzed the most abundant of the more than 370 NAD(P)H-dependent enzymes in *T. gondii*-infected HFF cells (Fig. S13) (20): i.e., the enzymes we assume will have an impact on the resulting fluorescence lifetime of NAD(P)H and will preferentially bind to the coenzymes (68). This time-course gene expression shows the most likely host enzymes to bind to NAD(P)H during *T. gondii* infection (Fig. S13), which are similar to the NAD(P)H-bound enzymes found in other parasites assessed using FLIM (68). We did not identify the host NADPH–oxidase complex, another NAD(P)H-dependent enzyme that plays an important role in *T. gondii* infection (69–71). Fourth, we analyzed the *T. gondii* gene expression of 58 genes related to redox biology (72) and found that *T. gondii* superoxide dismutase, catalase, and proteins with thioredoxin domains are upregulated during 48 HPI (Fig. S14). Fifth, we assessed the expression of other genes that should also be related to redox biology in *T. gondii* metabolism (Fig. S15) where *T. gondii* lactate dehydrogenase 1 and *T. gondii* glucose −6 phosphate dehydrogenase putative 2 are upregulated during the 48 HPI time course.

## DISCUSSION

**Fig 8:**
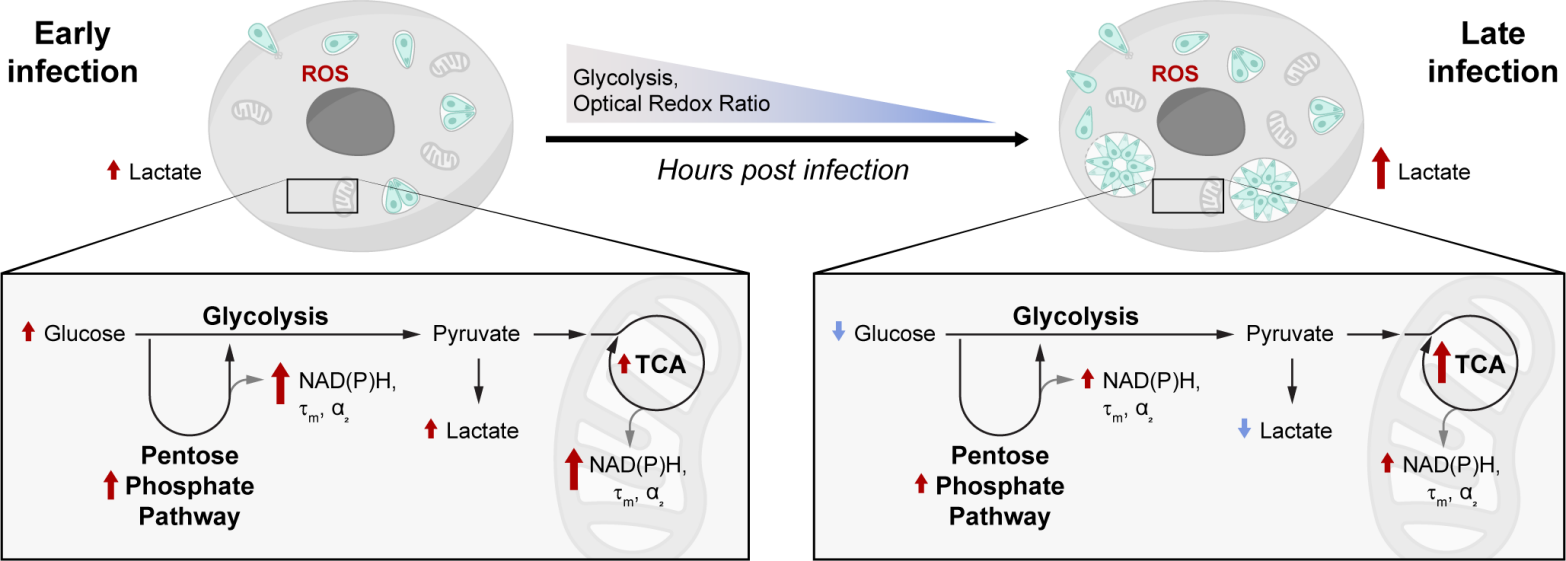
Summary of metabolic changes in ME49 *Toxoplasma gondii-*infected HFF cells. During ME49 *T. gondii* early infection, the host intracellular glucose increases and the host glycolytic, PPP, and TCA enzymes are upregulated; these three metabolic processes produce NAD(P)H. NAD(P)H mean lifetime (τ_m_) and the percentage of protein-bound NAD(P)H (α_2_) are elevated with early infection. Reactive oxygen species (ROS) are increased; the optical redox ratio (ORR) as well as host glycolysis decrease in infected cells over time. During late infection, the host intracellular glucose as well as the host glycolytic and PPP enzyme gene expression decrease, while some TCA cycle enzymes are still upregulated. ROS fluctuates over time, and the ORR and host glycolysis are low in infected cells over time. The lactate in media increases over time. Many host and parasite genes (described in Fig. S11 to S17) could be related to these changes in metabolism, especially those implicated in ROS production, glycolysis, TCA cycle, and PPP. *T. gondii-*infected HFF cells show an increase in the relative amount of bound NAD(P)H (α_2_) with respect to free NAD(P)H (α_1_) over the time course of infection. This abundance of bound NAD(P)H (α_2_) in *T. gondii*-infected HFF cells could be correlated to upregulated expression of enzymes that bind to NAD(P)H in *T. gondii* infection (Fig. **S13**) or to the abundance of PPP and the high rate of glycolysis in the intracellular ME49 *T. gondii* parasite.

Changes in host cell metabolism as a consequence of nutrient scavenging by intracellular parasites are difficult to study within living cells over time (19). This study sheds light on previously unexplored changes in host cell metabolism induced by *T. gondii* infection using noninvasive, label-free autofluorescence imaging. Most notably, OMI revealed shifts in host cell redox balance with *T. gondii* infection (modeled in Fig. 8), in particular a greater degree of oxidation in infected cells (Fig. 2). This change, represented by a decreased ORR, could be related to intracellular lactate levels, α-ketoglutarate dehydrogenase enzyme gene expression (Fig. S16A), glycolytic capacity (Fig. S16B), or a progressive decrease in glycolysis (Fig. 5A and 6C ; Fig. S16B) in *T. gondii*-infected cells, among other factors (Fig. 8). The changes in NAD(P)H and FAD lifetimes revealed by FLIM could be due to the effect of infection on host glycolysis, PPP, and TCA cycle (20). Our results also indicated the stimulation of metabolic pathways involving participation by protein-bound FAD (Fig. S8). While this phenomenon has been poorly investigated in *T. gondii* infection, it probably involves fatty acid beta-oxidation and the TCA cycle (73). Prior work indicates that the PPP may be the main producer of NAD(P)H in the *T. gondii* parasite, with *T. gondii* glucose-6-phosphate 1- dehydrogenase 2, an enzyme involved in the first step in the PPP, playing an important role in maintaining the cytosolic NADP/NADPH balance and tachyzoite antioxidant response (74).

Several reasons could underlie why we observe a greater proportion of protein-bound NAD(P)H in *T. gondii*-infected cells compared with uninfected cells (Fig. 3C). This observation trends with the gene expression of several host glycolytic enzymes (see Fig. S16A and S17), the TCA enzyme isocitrate dehydrogenase 3 a, and the PPP host enzyme glucose-6-phosphate dehydrogenase (Fig. S13 and S16A) (75–77). Thus, in our study, the increase in the proportion of protein-bound NAD(P)H upon *T. gondii* infection could be due to protein-bound NAD(P)H lifetime being sensitive to multiple fates of glucose carbon (78) (Fig. 8; Fig. S16B); the binding of NAD(P)H to different enzymes as discussed previously (Fig. 3; Fig. S13 and S16A); enzymes involved in the production of NAD(P)H such as glyceraldehyde-3-phosphate dehydrogenase (79) (Fig. S16A); or host immune response mechanisms to eliminate the parasite (80). Consistent with our results, previous FLIM measurements found increases in the proportion of bound NAD(P)H in human cells infected with the intracellular bacteria *Chlamydia trachomatis* (34), indicative of host cell starvation.

A key finding in this study is that *T. gondii* kiss and spit decreases the ORR and increases the proportion of protein-bound NAD(P)H (Fig. 4) in a similar trend to *T. gondii* full infection in HFF cells (Fig. 2; Fig. S9). Our whole cell mask and host cell mask analyses showed similar results, confirming that the parasite induced metabolic changes in the host cell even without full invasion (Fig. 4; Fig. S7). These findings are consistent with those of our previous work, demonstrating that kiss and spit alters PPP (producer of NADPH) and glycolysis and/or TCA cycle (producers of NADH) (21), as well as a prior study demonstrating that *T. gondii* manipulates uninfected–injected (or “kiss and spit”) cells much like infected–injected cells in an *in vivo* mouse model (39).

To understand the molecular mechanisms behind the NAD(P)H and redox changes observed through autofluorescence imaging, we also measured intracellular and extracellular glucose and lactate, extracellular flux, and ROS production. Glucose is the preferred nutrient for *T. gondii,* and its assimilation via glycolysis supports the optimal growth of the parasite (42, 81). Consistent with prior studies (42), our extracellular flux analysis detected high glycolytic activity in ME49 *T. gondii*-infected cells at early timepoints (Fig. 6C). Many studies have demonstrated that *T. gondii* can, however, propagate in the absence of glucose, using glutamine, lactate, or acetate as alternative sources of energy (42, 43). In this study, we found a decrease of intracellular glucose in *T. gondii-*infected host cells and an increase of lactate exportation over time (Fig. 5), which trends with the oxidized ORR quantified by OMI (Fig. 2). Similar results were observed in myoblasts and myotubes infected with *T. gondii* type II (82). Our previous metabolomic analysis (20) and the upregulated gene expression of glycolytic enzymes (19, 20) are also consistent with our current results (Fig. S17). Thus, *T. gondii* and host LDH activities are upregulated and trend with the intracellular measurements of glucose and lactate during infection (Fig. S17J) (19, 20). Pyruvate and lactate metabolites are abundant in *T. gondii* infection because the parasite must maintain pyruvate homeostasis (45). Lactate serves as a major circulating carbohydrate fuel (83), is exported from the parasite (84), and helps regulate the redox balance in infection, cancer, and immune cells (85–87). Low-glucose and high-lactate environments are immunosuppressive; these conditions are found in the placenta, gastrointestinal tract, and in the tumor microenvironment (85), as well as *T. gondii* infection (Fig. 5 and 8 ).

Prior work has found that ROS production fluctuates in host cells infected with *T. gondii* because of the oxidative stress from the host response, likely an effort to kill the parasite or induced by the parasite (64–66). Our finding that ROS production in infected cells fluctuated over time (Fig. 7) trends with the host gene expression of the DUOX1 enzyme in ME49 *T. gondii*-infected HFF cells (Fig. S13), suggesting that ROS production in these cells may impact the ORR and NAD(P)H lifetimes (Fig. 8). Elevated ROS levels have been found in *T. gondii*-infected host cells (64, 69, 82), in asymptomatic *T. gondii*-seropositive cats (88), and in human patients infected with *T. gondii* (89). The host ROS production can not only inhibit *T. gondii* growth (90) but also contributes to oxidative injury inflicting tissue damage and disease pathology (64, 66). Previous work has also suggested that redox and ROS changes in host cells can influence *T. gondii* differentiation from the tachyzoite to bradyzoite stage (82). Tachyzoite is a rapidly dividing asexual stage of *T. gondii* that produces acute infection in the host, and it is the *in vitro* culture stage used in this research. Then, the tachyzoite differentiates to a slower-growing encysted bradyzoite (91). In our follow-up studies, we will use OMI to study the metabolic switch from the tachyzoite to bradyzoite stage.

Applications of OMI, including this study, are currently limited by two primary obstacles. First, the conversion of NAD(P)H fluorescence intensity values to absolute concentration values is not straightforward because the different quantum yields of free and protein-bound NAD(P)H must be calculated (34, 92, 93). Second, NADH and NADPH are spectrally indistinguishable and have similar fluorescence lifetimes, so it is difficult to separate their contributions to the overall fluorescence signal. Although estimations of cellular concentrations suggest that a substantial part of the cellular fluorescence originates from NADH rather than from NADPH (94, 95), it could vary among cell types or disease state. Notwithstanding these limitations, metabolic profiling of infected cells by OMI will complement established large-scale genomic, transcriptomic, proteomic, and metabolomics approaches in the process of understanding how intracellular pathogens interfere with host cell metabolism. A more detailed understanding of the metabolic activity and needs of *T. gondii* during the intracellular growth phase is needed to conceive novel therapeutic strategies that target the pathogen in that phase without affecting the host. We expect the advancement of OMI will contribute to this understanding. OMI may be broadly used to investigate subcellular metabolic patterns, different strains of parasites, as well as differences in infection with different kinds of host cells.

## MATERIALS AND METHODS

### *T. gondii* strains and cell culture

Low passage mCherry type II-ME49 *T. gondii* was used for OMI. Low passage type II ME49 *T. gondii* was used for the rest of the experiments. The parental strain RHΔKU80 (RH) and the modified RHΔKU80ΔMAF (RHΔMAF) strains obtained from Dr. Boothroyd were used as controls for extracellular flux and ROS analysis. Human foreskin fibroblast (HFF) cells were used in this metabolomic study because the cells reach 100% confluency, are contact-inhibited, and quiescent in 2 weeks (96, 97). HFF cells were grown in DMEM with 10% fetal bovine serum (FBS), 2 mM L-glutamine, and 1% penicillin–streptomycin (Sigma-Aldrich). Once HFF cells were in deep quiescence, defined as 10 days post-confluency, Dulbecco’s modified Eagle medium (DMEM) was changed to metabolomic media (RPMI 1640 supplemented with 2 mM L-glutamine, 1% FBS dialyzed against PBS (MW cutoff of 10 kD), 10 mM HEPES, and 1% penicillin–streptomycin) for all metabolomic analyses.

### Time course of infection and kiss and spit

For OMI, HFF dishes in metabolic media and in triplicate were treated as follows: (a) uninfected; (b) infected with 2 × 10^6^ ME49 *T. gondii* tachyzoites; (c) infected with 2 × 10^6^ ME49 tachyzoites that had been preincubated with 1.5 µM cytochalasin D during 15 minutes at 37°C (Sigma-Aldrich); (d) incubated with 1.5 µM cytochalasin D (Sigma-Aldrich). Cytochalasin D was kept throughout the length of experiments and imaging.

### Two-photon imaging

Fluorescence lifetime images were taken on a custom-built inverted multiphoton microscope (Bruker Fluorescence Microscopy, Middleton, WI, USA), as previously described (98–100). The system consists of an ultrafast laser (Spectra Physics, Insight DS-Dual, Milpitas, CA, USA), an inverted microscope (Nikon, Eclipse Ti, Tokyo, Japan), and a 40 × water immersion (1.15 NA, Nikon) objective. NAD(P)H and FAD images were acquired sequentially for the same field of view using an excitation wavelength of 750 nm and a 440-/80-nm emission bandpass filter for NAD(P)H fluorescence and an excitation wavelength of 890 nm and a 550-/100-nm emission bandpass filter for FAD fluorescence. mCherry *T. gondii* was excited at 1,090 nm with emission at 690/50 nm. During imaging, dishes were maintained at 37°C and 5% CO_2_ using a stage-top incubator system (Tokai Hit). Fluorescence lifetime images were collected using time-correlated single-photon counting electronics (SPC-150, Becker and Hickl, Berlin, Germany) and a GaAsP photomultiplier tube (H7422P-40, Hamamatsu Photonics, Hamamatsu, Japan). A pixel dwell time of 4.8 µs was used to acquire 256 *×* 256-pixel images over 60 second total integration time. The photon count rates were maintained at 1–2 *×* 105 photons/second to ensure adequate photon observations for lifetime decay fits, and no photobleaching was observed. The instrument response function was measured from the second harmonic generation of urea crystals excited at 900 nm, and the full width at half maximum (FWHM) was calculated to be 220 ps.

### Quantification of fluorescence lifetime components

NAD(P)H and FAD fluorescence lifetime images were analyzed using SPCImage software (v8.1, Becker & Hickl, Berlin, Germany) as previously described (101). Fluorescence lifetime components were computed for each image pixel by fitting the pixel-wise decay curves to a biexponential model convolved with the instrument response function, using a weighted least squares algorithm. The two-component exponential decay model is $I\left(t\right)=\alpha_{1}e^{-t/\tau_{1}}+\alpha_{2}e^{-t/\tau_{2}}+C$, where *I(t)* is the fluorescence intensity at time *t* after the laser excitation pulse; τ_1_ and τ_2_ are the fluorescence lifetimes of the short and long lifetime components, respectively; α_1_ and α_2_ are the fractional contributions of the short and long lifetime components, respectively; and *C* accounts for background light (30, 101). A two-component model was used because both NAD(P)H and FAD can exist in two conformational states, bound or unbound (32, 102). We use a binning factor of 1 or 2.

### Creating HFF whole-cell masks

Initial whole-cell masks were created manually using the software CellProfiler. Whole cell was defined as the cell border including the nuclei. These masks were manually revised as needed using the Napari image viewer.

### Calculating optical redox ratio, NAD(P)H, and FAD parameters

Single-cell OMI parameters were calculated with a custom Python library, *cell-analysis-tools* (103) using SPCImage fluorescence decay fits, and manually corrected whole cell masks. The ORR (calculated as $I_{NAD\left(P\right)H}/\left(I_{NAD\left(P\right)H}+I_{FAD}\right)$) and FLIM fit parameters for NAD(P)H and FAD (τ_1_, τ_2_, α_1,_ α_2,_ and τ_m_, defined as α_1_ τ_1_ + α_2_ τ_2_) were extracted for each cell (Table 1).

### Quantifying *Toxoplasma gondii* in mCherry images

In Python, the mCherry intensity image was loaded, and the intensity of the top 5% of the pixels was mapped to equal the 95-percentile intensity value, and then from the resulting image, the top 10% brightest pixels of the image were kept (Fig. S1). The resulting image was then binarized and then dilated using an octagon with footprint (1, 1). Binarization was done by making all pixels with values greater than 0 or equal to 1. Holes in the connected components were filled using the *binary_fill_holes* function from the image processing library *scikit-image* (104), followed by the function *remove_small_objects* from the same library to remove small sections of connected components less than 30 pixels in area. Another version of the mask was then created by taking the original NAD(P)H intensity image and keeping the 5% brightest pixels. Lastly, these two images were combined using a bitwise OR operation to create the final binary mask (Fig. S1).

### Quantifying intracellular *Toxoplasma* and establishing a 5% threshold

The percentage of *T. gondii* infection in each cell was quantified by first multiplying the final *T. gondii* mask by the final whole-cell mask to assign *T. gondii* pixels to individual cells. The sum of pixels in this resulting intracellular *T. gondii* mask was divided by the total number of pixels in the whole-cell mask (Fig. S2A).

### Metabolic profiling with the Seahorse XFp extracellular flux analyzer

HFF cells were seeded in a Seahorse 96-well plate and allowed to reach confluency and quiescence for 2 weeks. All subsequent steps were performed in metabolic media. Cells were infected with 6 × 10^3^ tachyzoites/well from the three different strains of *T. gondii*: ME49, RHΔMAF, and RHΔKU80 (RH) plus an uninfected HFF control group. The Cell Mito Stress Test was performed using 1 µM oligomycin, 2 µM carbonyl cyanide-4 phenylhydrazone (FCCP), and 0.5 µM rotenone. The glycolysis stress test was performed using 10 mM glucose, 2 µM oligomycin, and 50 mM 2-DG. The oxygen consumption rate (OCR) and extracellular acidification rate (ECAR) were measured using a Seahorse Bioscience XF96 Extracellular Flux Metabolic Analyzer in the Small Molecule Screening Facility at the University of Wisconsin. Graphs and statistical analysis were performed in Wave v 2.6.3.5 (Agilent), and statistical analysis was performed in GraphPad Prism.

### ROS labeling

HFF cells were seeded in a 24-well plate and allowed to reach confluency and quiescence for 2 weeks. Cells were infected with 2 × 10^5^ tachyzoites/well from the three different strains of *T. gondii*: ME49, RHΔMAF, and RHΔKU80 (RH) plus an uninfected HFF control group. Then, HFF cells were stained with 5 µM CellROX Green Reagent (Molecular Probes, Eugene, USA) in complete medium for 30 minutes at 37°C and 5% CO_2_. Fluorescence was measured in an IncuCyte S3 Live-Cell Analysis System.

### Lactate and glucose assays

Quiescent HFF cells in a 24-well plate were infected with 2 × 10^5^ ME49 tachyzoites/well in metabolic culture media. Extracellular and intracellular samples were collected from three biological replicates for each of the seven timepoints post-infection. Samples were homogenized by sonication for intracellular assays, and Abcam glucose (ab65333) and lactate (ab65330) assay kits were used. Samples were read in a plate reader at 533/587 nm, and concentrations in pMol were determined by standard glucose and lactate curves with standards.

### Gene expression analysis

We re-analyzed our previous published gene expression analysis (accession number PRJNA497277) from ME49 *T. gondii*-infected HFF cells (20). Lists of significant genes were used as input for gene ontology enrichment analysis using the Database for Annotation, Visualization, and Integrated Discovery (DAVID, v6.8) (67). First, we performed clustering by gene ontology term of significant genes during each timepoint. Second, we performed clustering by significant genes related to the reactome during each timepoint. Third, we generated heatmaps of time-course gene expression of the most likely host enzymes that bind to NAD(P)H during *T. gondii* infection, as have been published previously for other parasites assessed by FLIM (68). Fourth, we analyzed the expression of 58 genes related to redox biology in *T. gondii.* Fifth, we analyzed the expression of other genes related to redox biology in *T. gondii.*

### Statistical analyses

Biological repeats are defined as separate infections timepoint collected on 2 separate days. Graphs were generated using open-source Python packages Matplotlib and Seaborn (105). Each data point represents a single cell, and boxplots show the median and 95% CI. OMI statistical analysis was performed using the statannotations Python package (106). GraphPad Prism 10.0.0 was used to create graphs and run statistical analysis for glucose, lactate, and extracellular flux measures. For OMI data, the graphs represent the average of two independent experiments with different replicates. Thus, for the purpose of the analysis, every image was treated as an independent observation. Pairwise analysis was performed using a *t*-test adjusted for multiple comparisons with a Bonferroni correction. Multiple groups were compared using one-way ANOVA with post-hoc Tukey tests. We considered *P* < 0.05 to be significant, and all exact p-values are included in a supplemental spreadsheet.

## Data Availability

Codes used in this manuscript are available in https://github.com/skalalab/gallego_g-omi_toxoplasma_redox_ratio. Images are available upon request.

## SUPPLEMENTAL MATERIAL

Supplemental Figures mbio.00727-24-s0001.pdf Fig. S1-S17.

Supplemental Legends mbio.00727-24-s0002.docx Legends for the supplemental figures and the Excel spreadsheet.

Supplemental File mbio.00727-24-s0003.xlsx Statistical analysis of OMI.

ASM does not own the copyrights to Supplemental Material that may be linked to, or accessed through, an article. The authors have granted ASM a non-exclusive, world-wide license to publish the Supplemental Material files. Please contact the corresponding author directly for reuse.
