# Supplementary material for: Metabolic changes in Toxoplasma gondii-infected host cells measured by autofluorescence imaging
Source: mBio. 2024 Jul 8;15(8):e00727-24. doi: 10.1128/mbio.00727-24 (PMC11323734; doi:10.1128/mbio.00727-24)
Supplement: Supplemental Figures — Fig. S1-S17. [file mbio.00727-24-s0001.pdf]

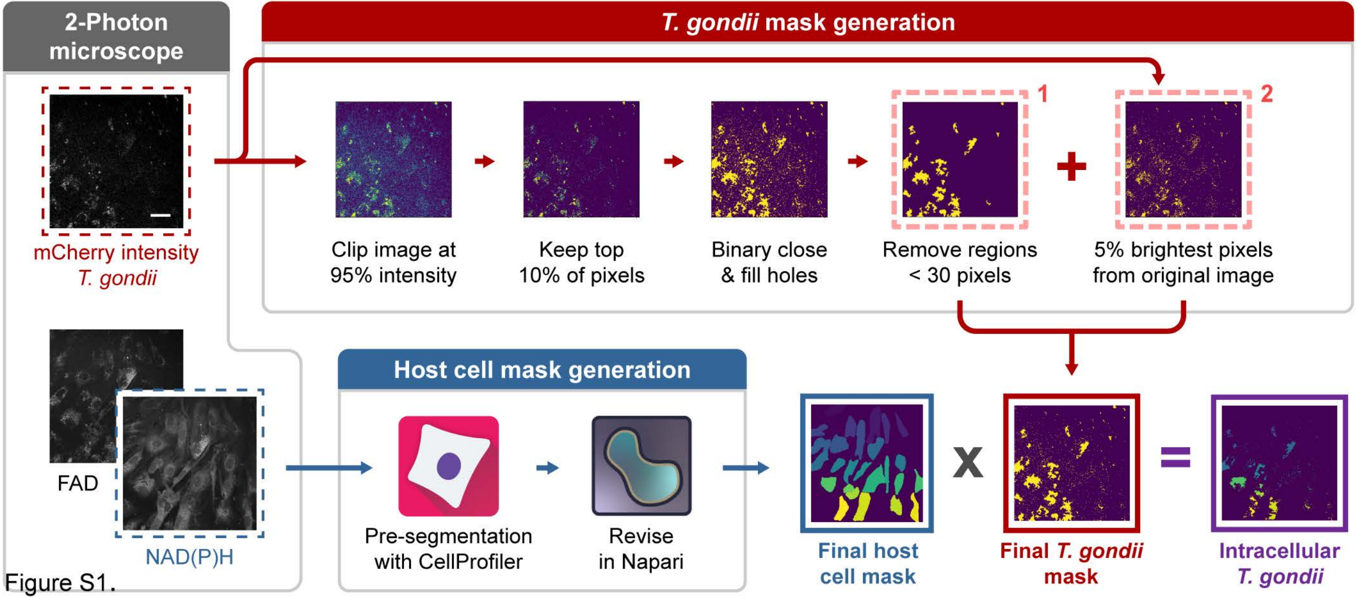
2-Photon microscope
mCherry intensity
T. gondii
FAD
NAD(P)H
T. gondii mask generation
Clip image at
95% intensity
Keep top
10% of pixels
Binary close
& fill holes
Remove regions
< 30 pixels
1
5% brightest pixels
from original image
2
Host cell mask generation
Pre-segmentation
with CellProfiler
Revise
in Napari
Final host
cell mask
X
Final T. gondii
mask
=
Intracellular
T. gondii

Figure S1.

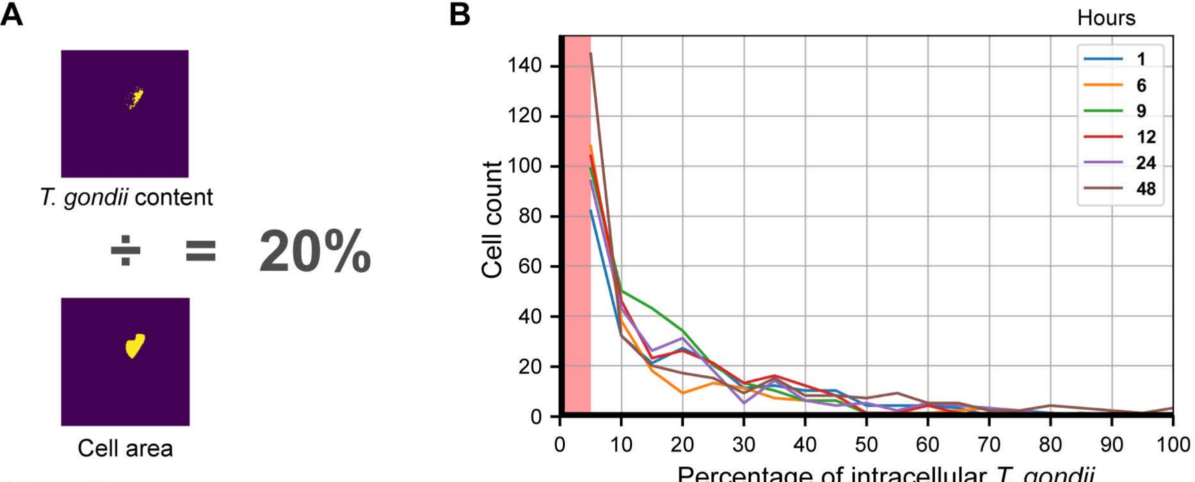
A
T. gondii content
÷
=
20%
Cell area
B
Hours
1
6
9
12
24
48
Cell count
0
20
40
60
80
100
120
140
0
10
20
30
40
50
60
70
80
90
100
Percentage of intracellular T. gondii

Figure S2.

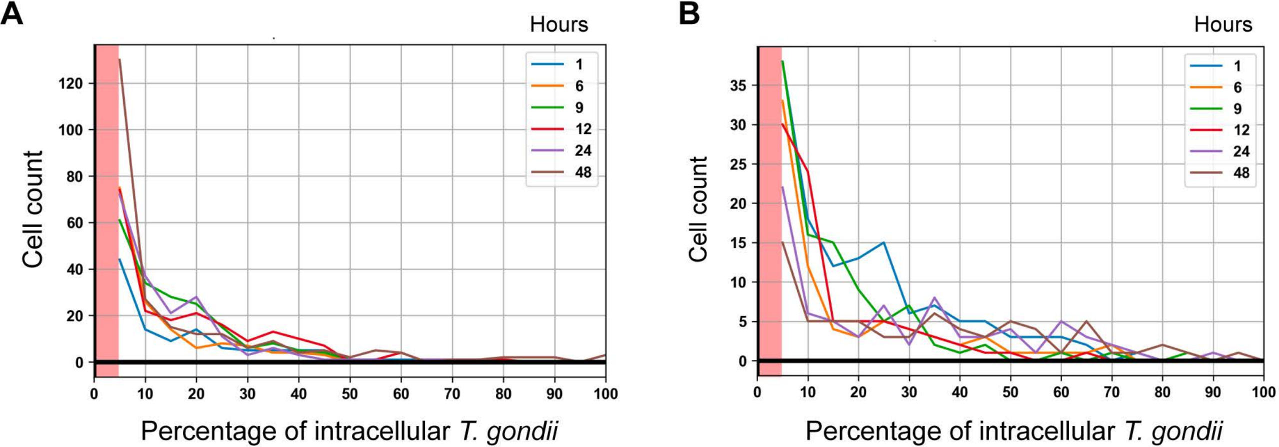
A
Hours
1
6
9
12
24
48
Cell count
0
20
40
60
80
100
120
0
10
20
30
40
50
60
70
80
90
100
Percentage of intracellular T. gondii
B
Hours
1
6
9
12
24
48
Cell count
0
5
10
15
20
25
30
35
0
10
20
30
40
50
60
70
80
90
100
Percentage of intracellular T. gondii

Figure S3.

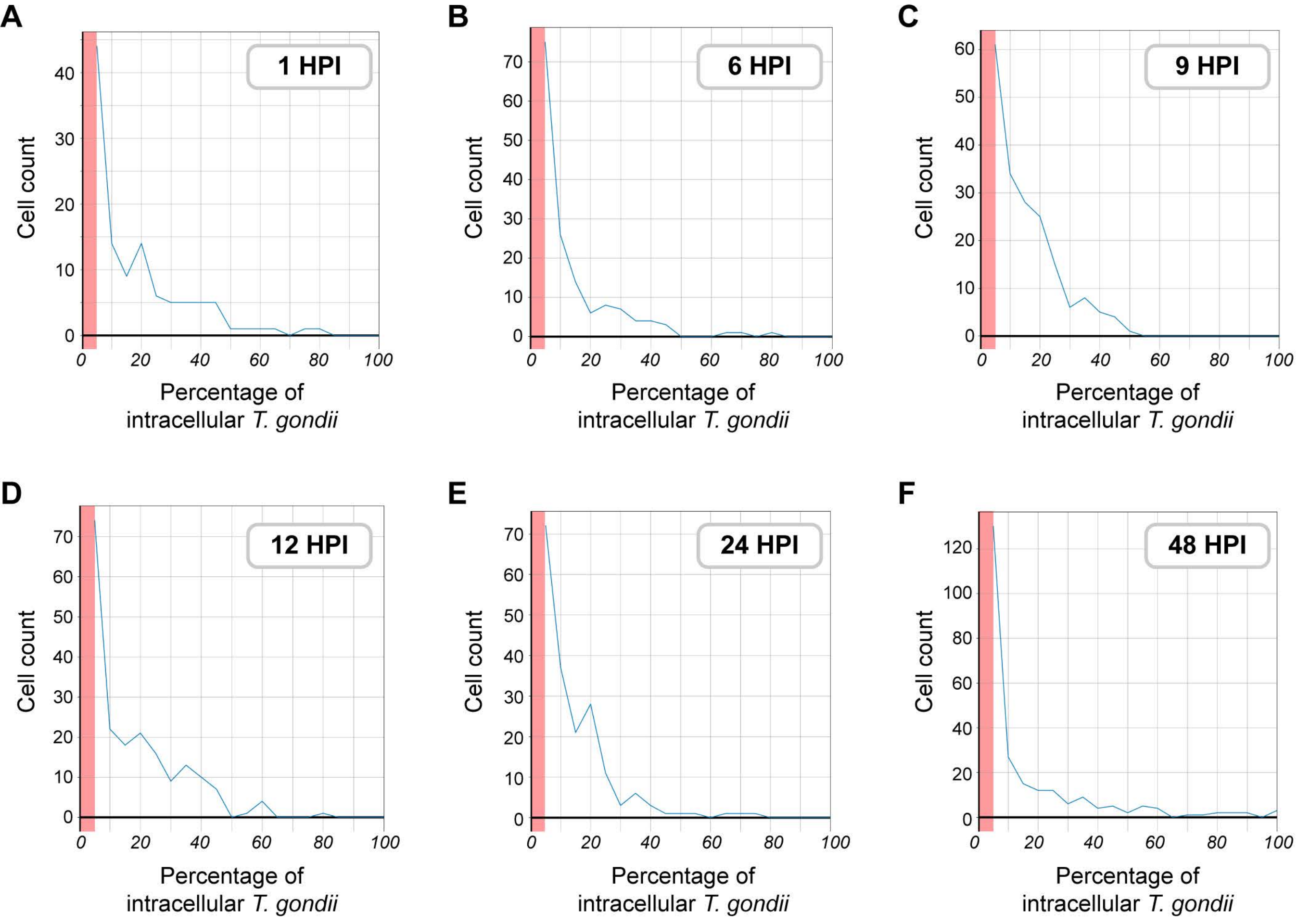
A
1 HPI
B
6 HPI
C
9 HPI
D
12 HPI
E
24 HPI
F
48 HPI
Cell count
Percentage of intracellular T. gondii

Figure S4.

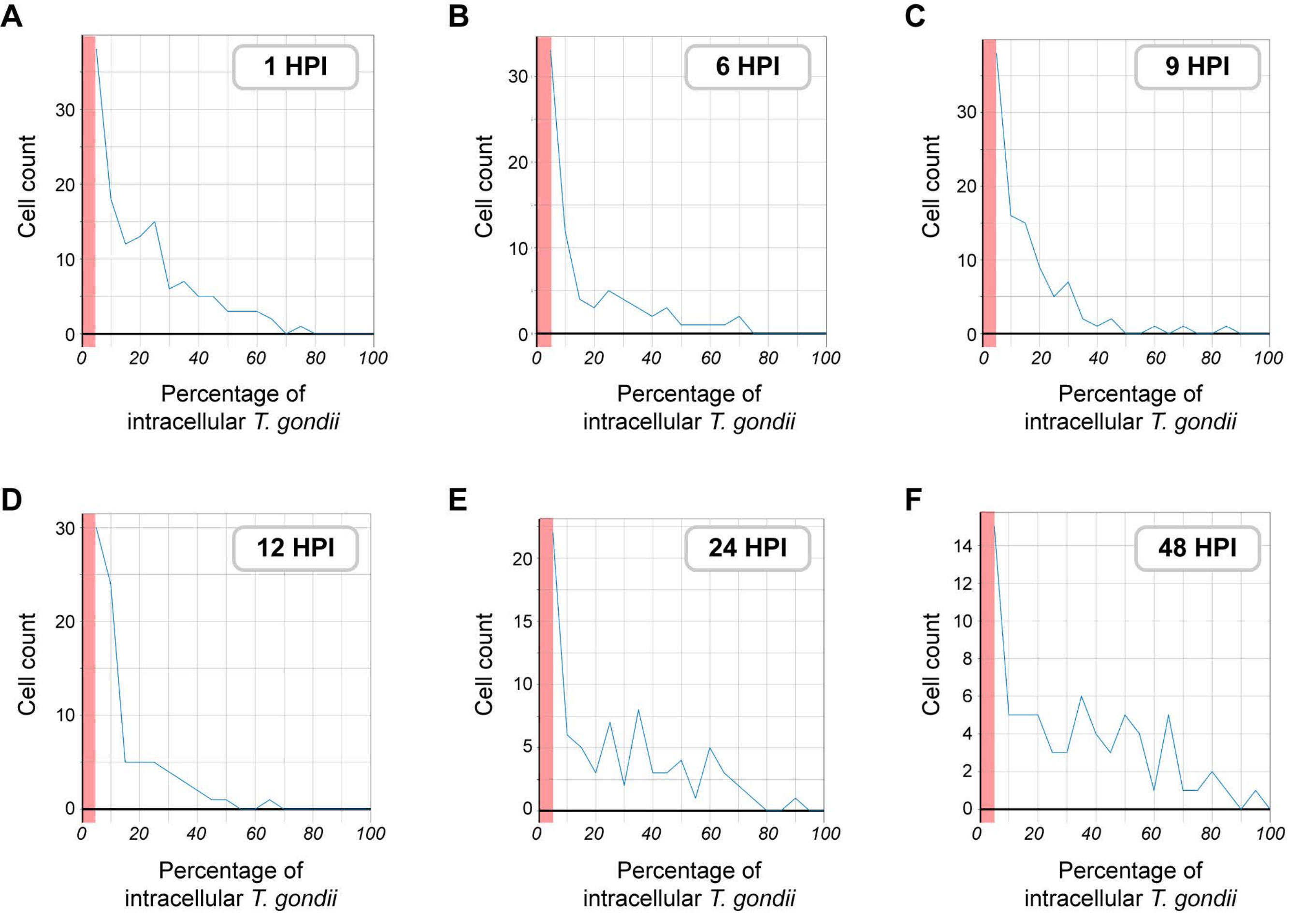
A
1 HPI
Cell count
Percentage of intracellular *T. gondii*
B
6 HPI
Cell count
Percentage of intracellular *T. gondii*
C
9 HPI
Cell count
Percentage of intracellular *T. gondii*
D
12 HPI
Cell count
Percentage of intracellular *T. gondii*
E
24 HPI
Cell count
Percentage of intracellular *T. gondii*
F
48 HPI
Cell count
Percentage of intracellular *T. gondii*

Figure S5.

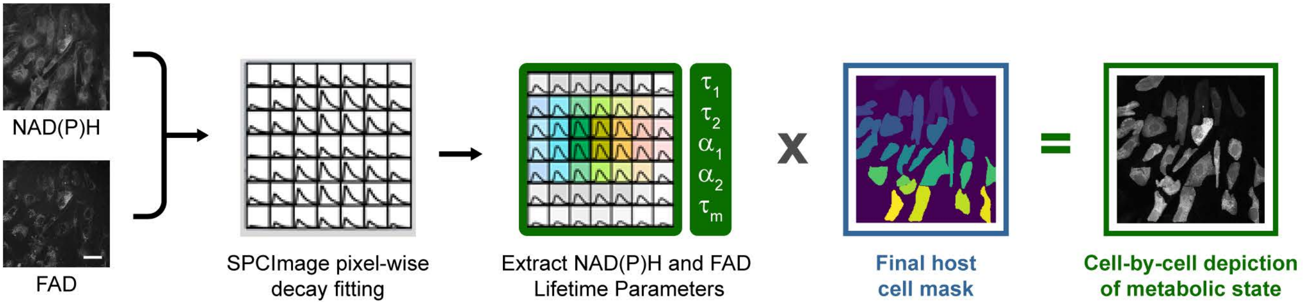

NAD(P)H
FAD
SPCImage pixel-wise decay fitting
τ_1
τ_2
α_1
α_2
τ_m
Extract NAD(P)H and FAD Lifetime Parameters
x
Final host cell mask
=
Cell-by-cell depiction of metabolic state

Figure S6.

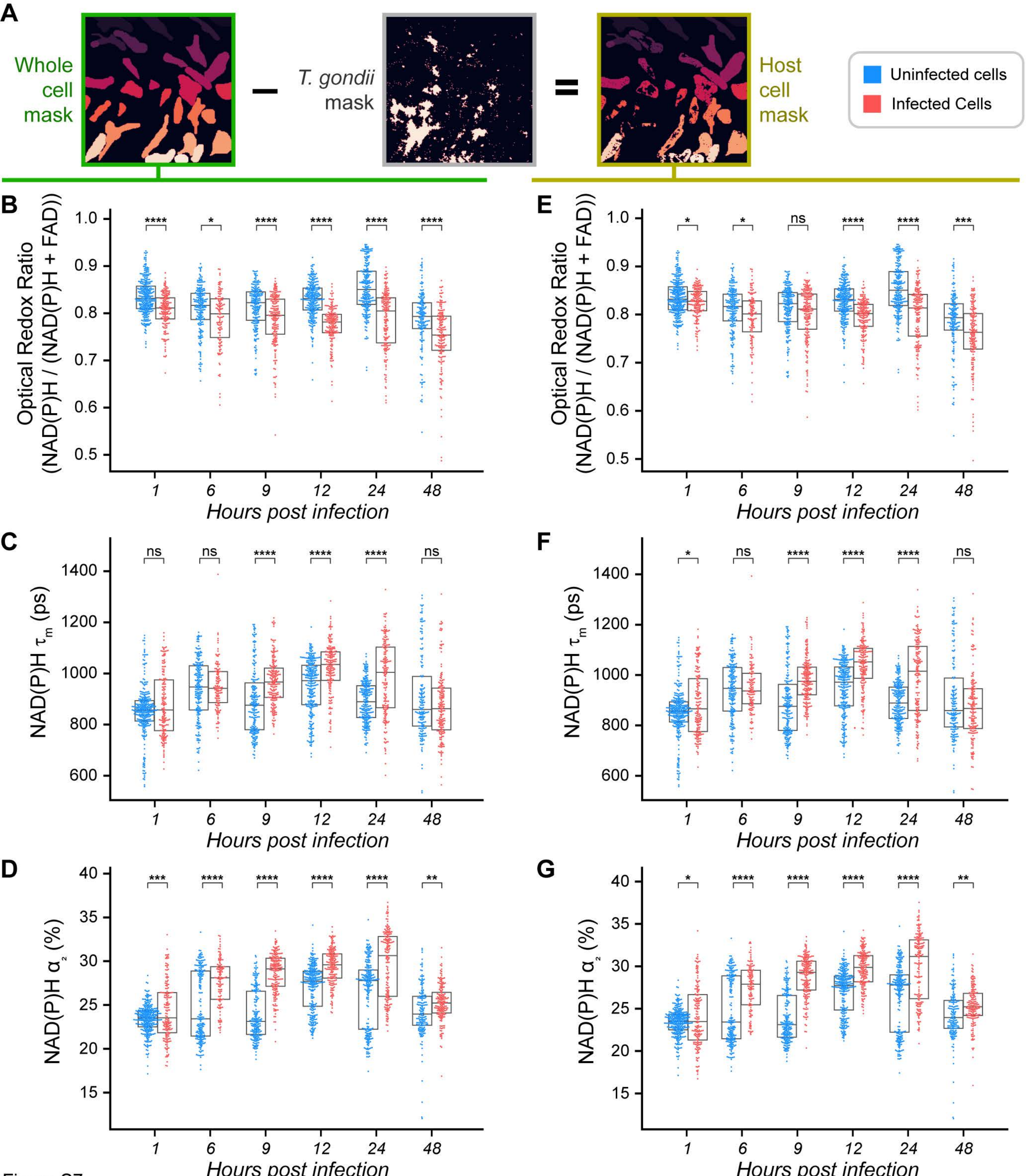
A
Whole cell mask
−
T. gondii mask
=
Host cell mask
Uninfected cells
Infected Cells
B
Optical Redox Ratio (NAD(P)H / (NAD(P)H + FAD))
**** * **** **** **** ****
Hours post infection
C
NAD(P)H τm (ps)
ns ns **** **** **** ns
Hours post infection
D
NAD(P)H α2 (%)
*** **** **** **** **** **
Hours post infection
E
Optical Redox Ratio (NAD(P)H / (NAD(P)H + FAD))
* * ns **** **** ***
Hours post infection
F
NAD(P)H τm (ps)
* ns **** **** **** ns
Hours post infection
G
NAD(P)H α2 (%)
* **** **** **** **** **
Hours post infection

Figure S7.

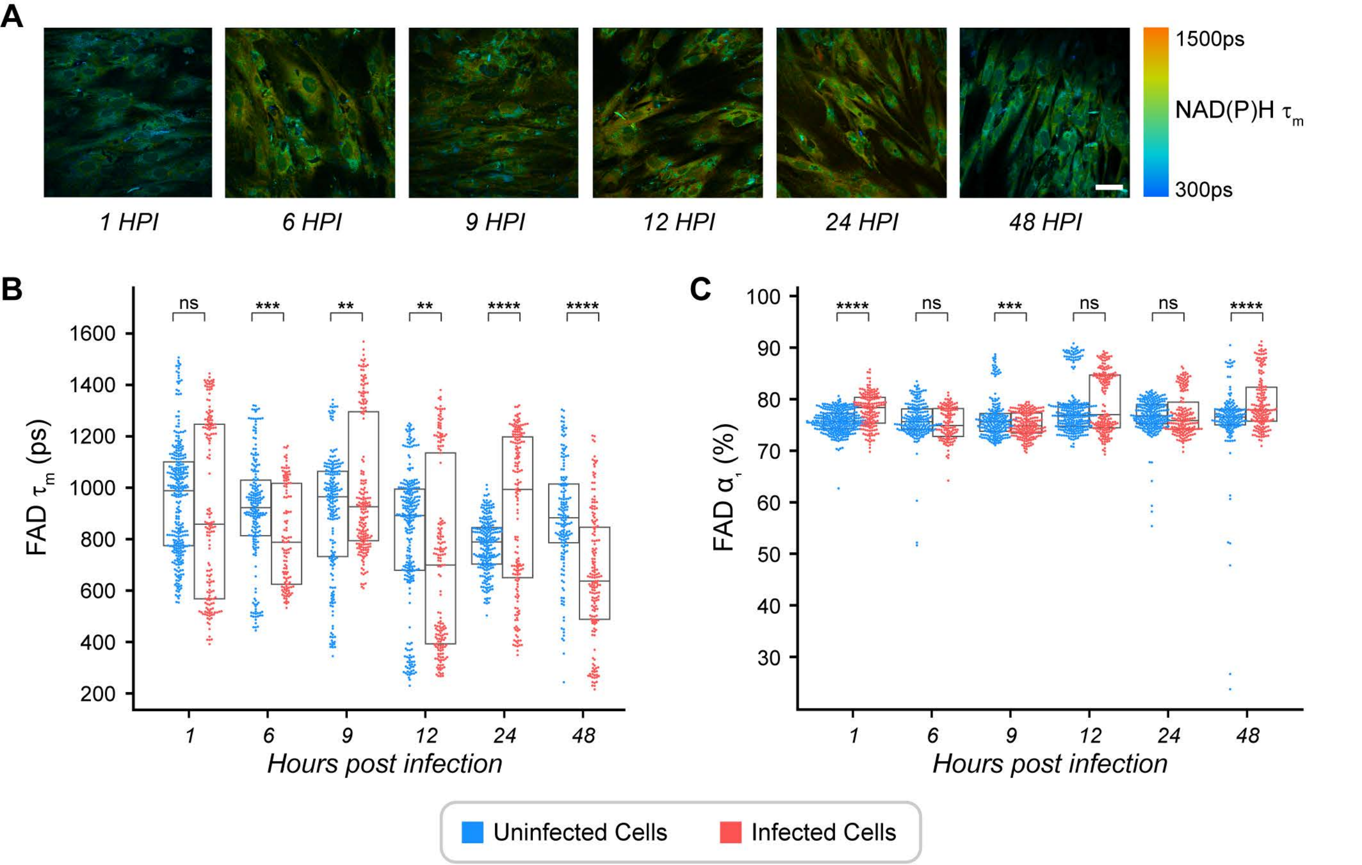

A
1500ps
NAD(P)H τ_m
300ps
1 HPI
6 HPI
9 HPI
12 HPI
24 HPI
48 HPI
B
FAD τ_m (ps)
ns
***
**
**
****
****
1600
1400
1200
1000
800
600
400
200
1
6
9
12
24
48
Hours post infection
C
FAD α_1 (%)
****
ns
***
ns
ns
****
100
90
80
70
60
50
40
30
1
6
9
12
24
48
Hours post infection
Uninfected Cells
Infected Cells

Figure S8.

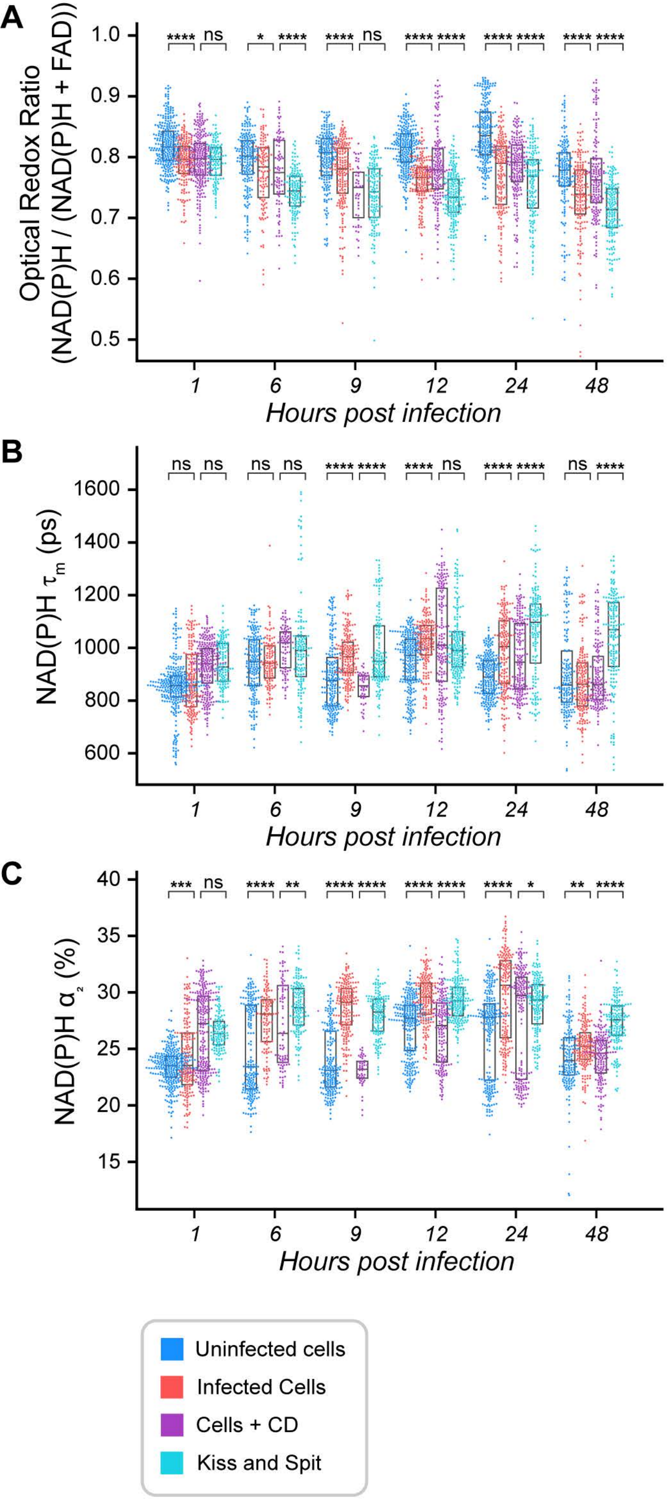
A
Optical Redox Ratio (NAD(P)H / (NAD(P)H + FAD))
1.0
0.9
0.8
0.7
0.6
0.5
**** ns * **** **** ns **** **** **** **** **** ****
1 6 9 12 24 48
Hours post infection
B
NAD(P)H τm (ps)
1600
1400
1200
1000
800
600
ns ns ns ns **** **** **** ns **** **** ns ****
1 6 9 12 24 48
Hours post infection
C
NAD(P)H α2 (%)
40
35
30
25
20
15
*** ns **** ** **** **** **** **** **** * ** ****
1 6 9 12 24 48
Hours post infection
Uninfected cells
Infected Cells
Cells + CD
Kiss and Spit

Figure S9.

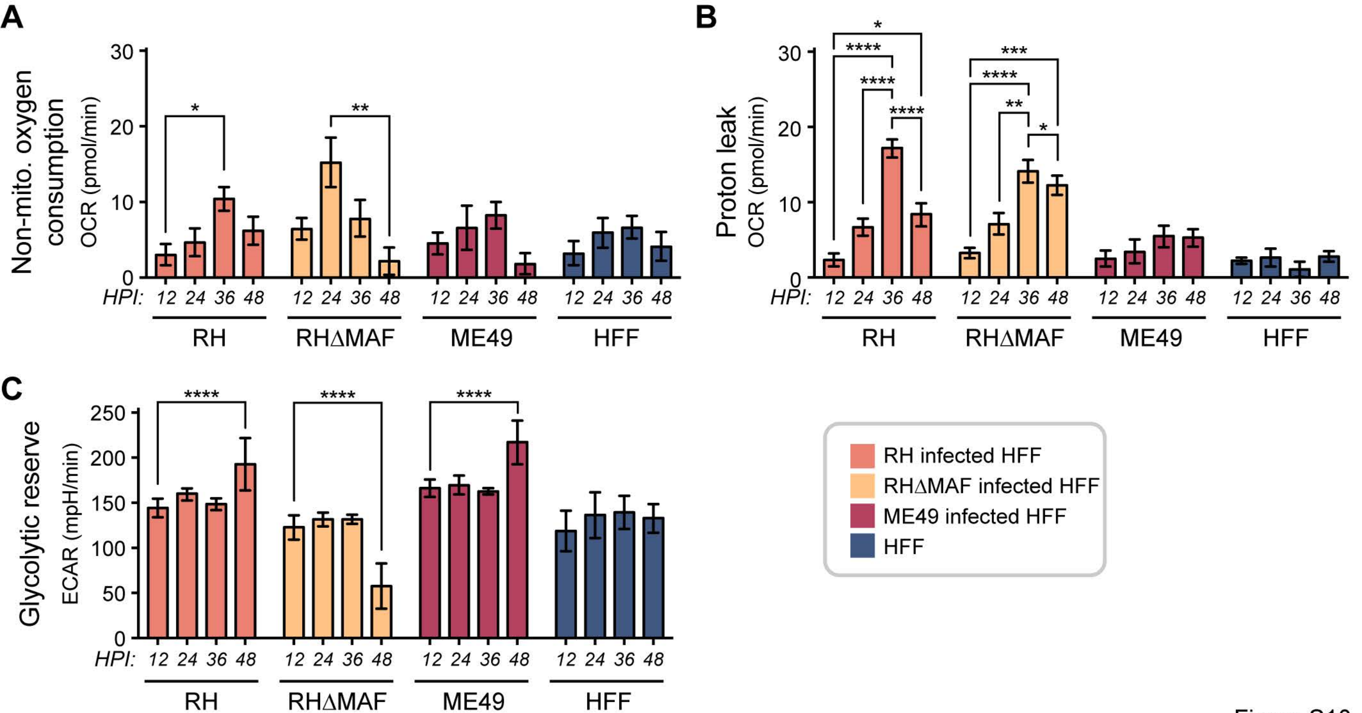

A
Non-mito. oxygen consumption
OCR (pmol/min)
30
20
10
0
HPI: 12 24 36 48
RH
RHΔMAF
ME49
HFF
B
Proton leak
OCR (pmol/min)
C
Glycolytic reserve
ECAR (mpH/min)
250
200
150
100
50
RH infected HFF
RHΔMAF infected HFF
ME49 infected HFF
HFF

Figure S10.

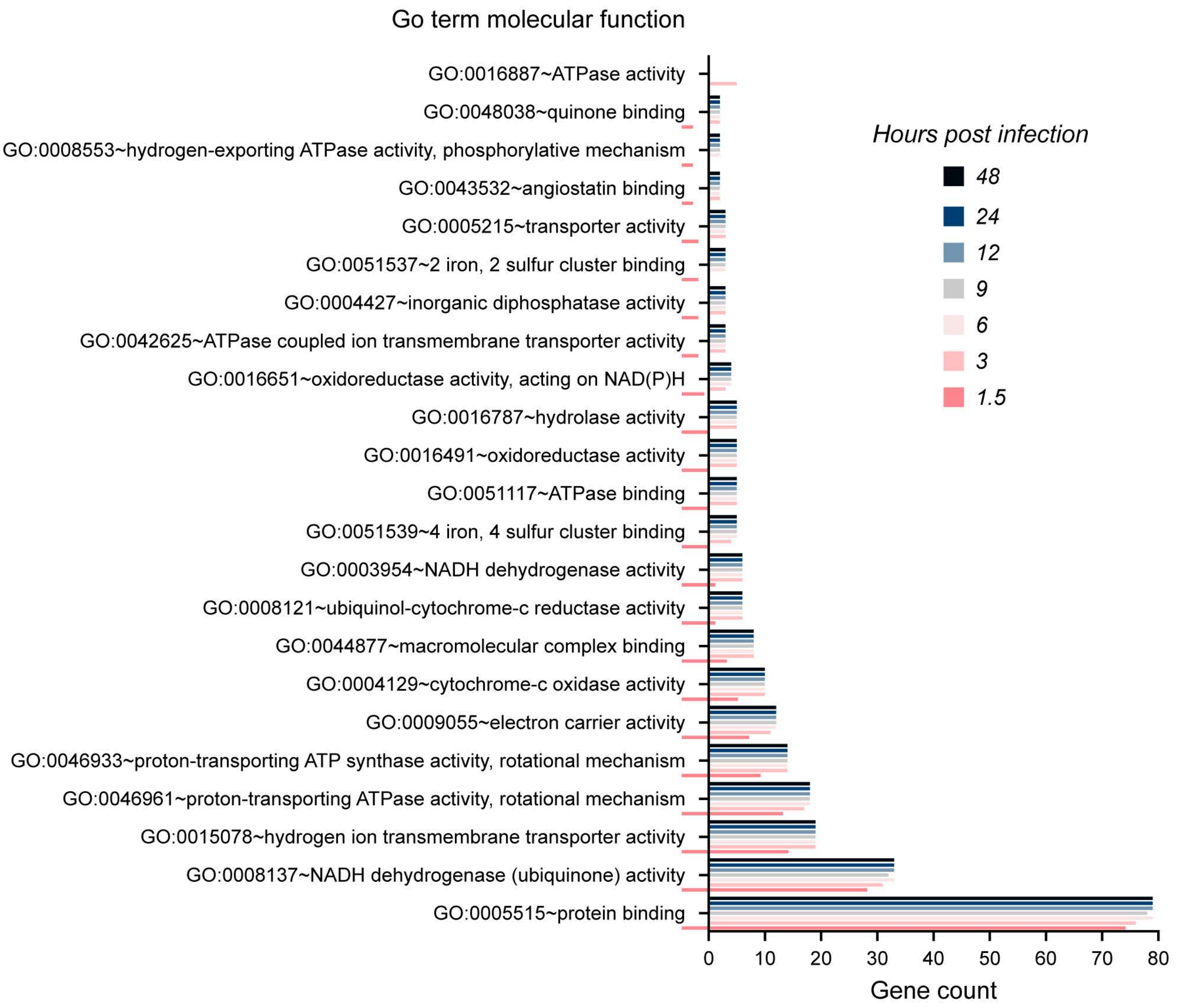
Go term molecular function
GO:0016887~ATPase activity
GO:0048038~quinone binding
GO:0008553~hydrogen-exporting ATPase activity, phosphorylative mechanism
GO:0043532~angiostatin binding
GO:0005215~transporter activity
GO:0051537~2 iron, 2 sulfur cluster binding
GO:0004427~inorganic diphosphatase activity
GO:0042625~ATPase coupled ion transmembrane transporter activity
GO:0016651~oxidoreductase activity, acting on NAD(P)H
GO:0016787~hydrolase activity
GO:0016491~oxidoreductase activity
GO:0051117~ATPase binding
GO:0051539~4 iron, 4 sulfur cluster binding
GO:0003954~NADH dehydrogenase activity
GO:0008121~ubiquinol-cytochrome-c reductase activity
GO:0044877~macromolecular complex binding
GO:0004129~cytochrome-c oxidase activity
GO:0009055~electron carrier activity
GO:0046933~proton-transporting ATP synthase activity, rotational mechanism
GO:0046961~proton-transporting ATPase activity, rotational mechanism
GO:0015078~hydrogen ion transmembrane transporter activity
GO:0008137~NADH dehydrogenase (ubiquinone) activity
GO:0005515~protein binding
Hours post infection
48
24
12
9
6
3
1.5
0
10
20
30
40
50
60
70
80
Gene count

Figure S11.

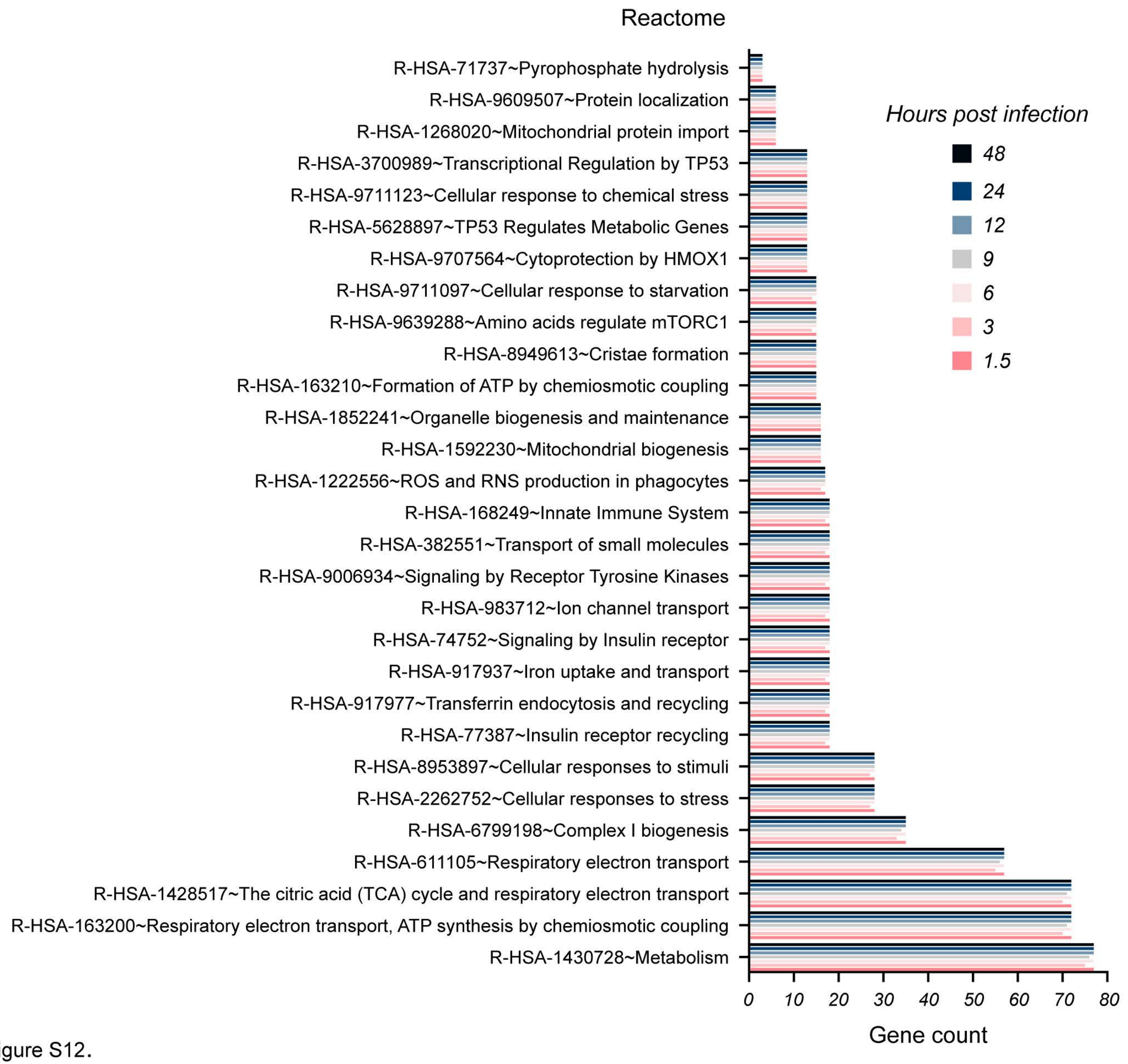
Reactome
R-HSA-71737~Pyrophosphate hydrolysis
R-HSA-9609507~Protein localization
R-HSA-1268020~Mitochondrial protein import
R-HSA-3700989~Transcriptional Regulation by TP53
R-HSA-9711123~Cellular response to chemical stress
R-HSA-5628897~TP53 Regulates Metabolic Genes
R-HSA-9707564~Cytoprotection by HMOX1
R-HSA-9711097~Cellular response to starvation
R-HSA-9639288~Amino acids regulate mTORC1
R-HSA-8949613~Cristae formation
R-HSA-163210~Formation of ATP by chemiosmotic coupling
R-HSA-1852241~Organelle biogenesis and maintenance
R-HSA-1592230~Mitochondrial biogenesis
R-HSA-1222556~ROS and RNS production in phagocytes
R-HSA-168249~Innate Immune System
R-HSA-382551~Transport of small molecules
R-HSA-9006934~Signaling by Receptor Tyrosine Kinases
R-HSA-983712~Ion channel transport
R-HSA-74752~Signaling by Insulin receptor
R-HSA-917937~Iron uptake and transport
R-HSA-917977~Transferrin endocytosis and recycling
R-HSA-77387~Insulin receptor recycling
R-HSA-8953897~Cellular responses to stimuli
R-HSA-2262752~Cellular responses to stress
R-HSA-6799198~Complex I biogenesis
R-HSA-611105~Respiratory electron transport
R-HSA-1428517~The citric acid (TCA) cycle and respiratory electron transport
R-HSA-163200~Respiratory electron transport, ATP synthesis by chemiosmotic coupling
R-HSA-1430728~Metabolism
Hours post infection
48
24
12
9
6
3
1.5
0
10
20
30
40
50
60
70
80
Gene count

Figure S12.

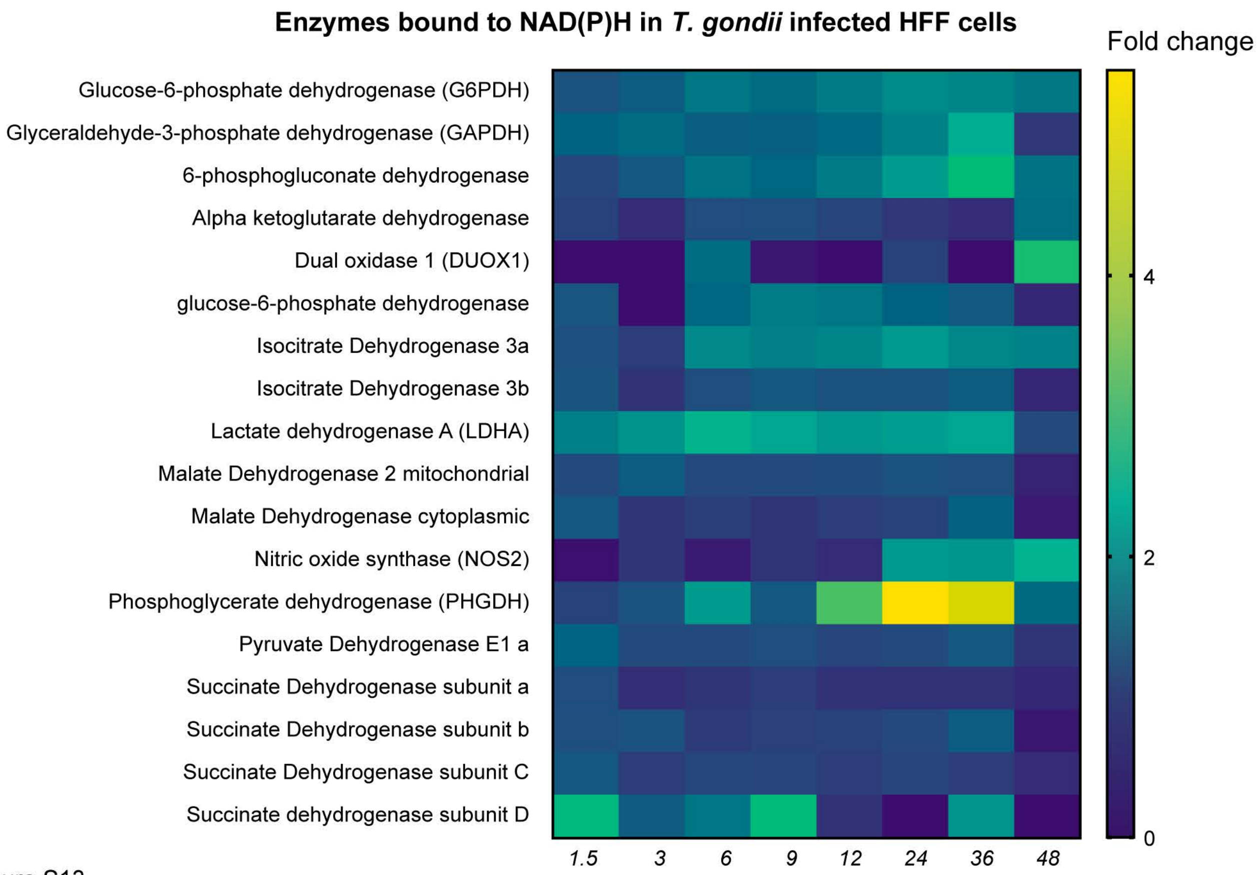

Enzymes bound to NAD(P)H in *T. gondii* infected HFF cells
Fold change
Glucose-6-phosphate dehydrogenase (G6PDH)
Glyceraldehyde-3-phosphate dehydrogenase (GAPDH)
6-phosphogluconate dehydrogenase
Alpha ketoglutarate dehydrogenase
Dual oxidase 1 (DUOX1)
glucose-6-phosphate dehydrogenase
Isocitrate Dehydrogenase 3a
Isocitrate Dehydrogenase 3b
Lactate dehydrogenase A (LDHA)
Malate Dehydrogenase 2 mitochondrial
Malate Dehydrogenase cytoplasmic
Nitric oxide synthase (NOS2)
Phosphoglycerate dehydrogenase (PHGDH)
Pyruvate Dehydrogenase E1 a
Succinate Dehydrogenase subunit a
Succinate Dehydrogenase subunit b
Succinate Dehydrogenase subunit C
Succinate dehydrogenase subunit D
1.5
3
6
9
12
24
36
48
4
2
0

Figure S13.

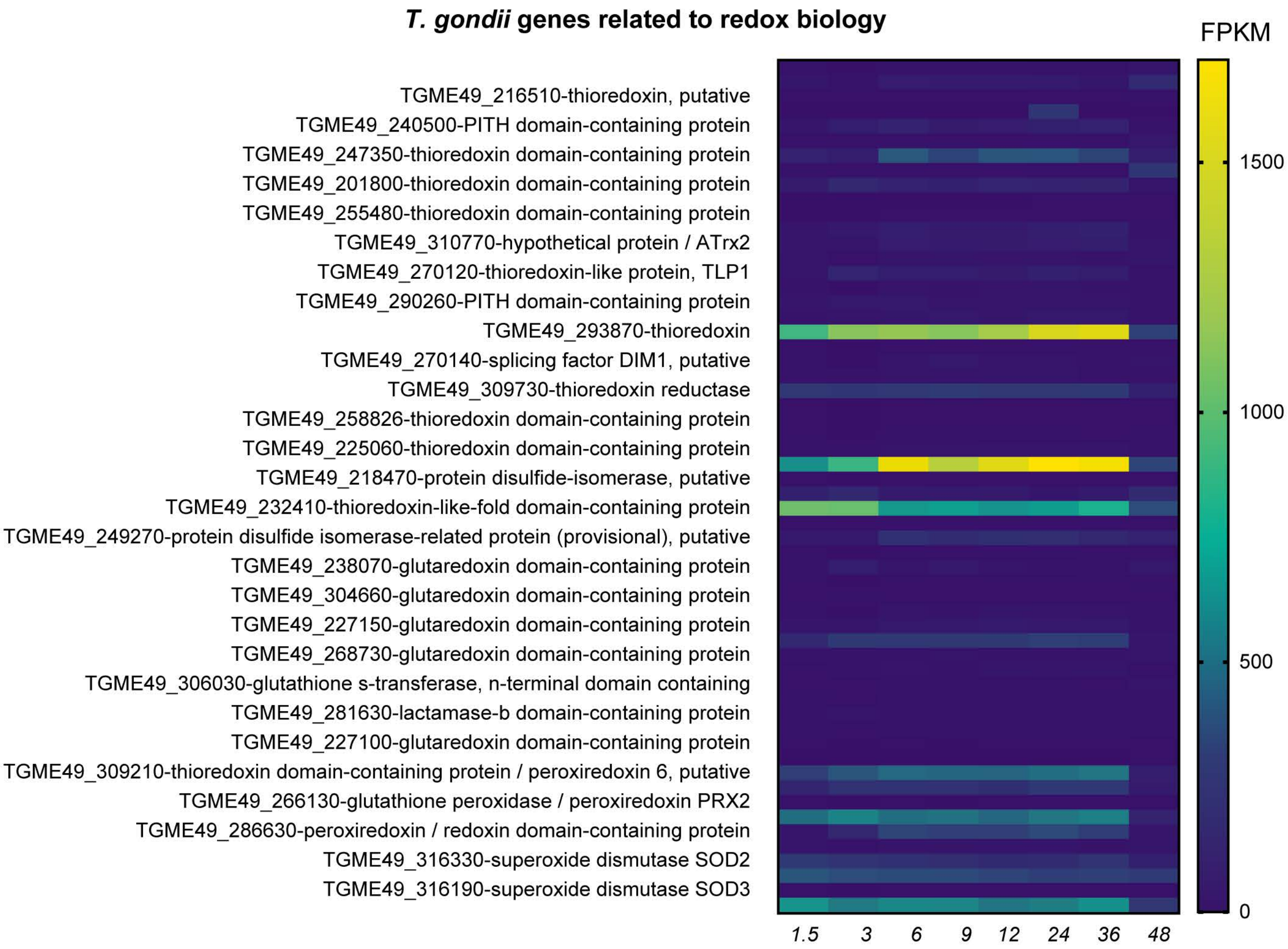

T. gondii genes related to redox biology
FPKM
TGME49_216510-thioredoxin, putative
TGME49_240500-PITH domain-containing protein
TGME49_247350-thioredoxin domain-containing protein
TGME49_201800-thioredoxin domain-containing protein
TGME49_255480-thioredoxin domain-containing protein
TGME49_310770-hypothetical protein / ATrx2
TGME49_270120-thioredoxin-like protein, TLP1
TGME49_290260-PITH domain-containing protein
TGME49_293870-thioredoxin
TGME49_270140-splicing factor DIM1, putative
TGME49_309730-thioredoxin reductase
TGME49_258826-thioredoxin domain-containing protein
TGME49_225060-thioredoxin domain-containing protein
TGME49_218470-protein disulfide-isomerase, putative
TGME49_232410-thioredoxin-like-fold domain-containing protein
TGME49_249270-protein disulfide isomerase-related protein (provisional), putative
TGME49_238070-glutaredoxin domain-containing protein
TGME49_304660-glutaredoxin domain-containing protein
TGME49_227150-glutaredoxin domain-containing protein
TGME49_268730-glutaredoxin domain-containing protein
TGME49_306030-glutathione s-transferase, n-terminal domain containing
TGME49_281630-lactamase-b domain-containing protein
TGME49_227100-glutaredoxin domain-containing protein
TGME49_309210-thioredoxin domain-containing protein / peroxiredoxin 6, putative
TGME49_266130-glutathione peroxidase / peroxiredoxin PRX2
TGME49_286630-peroxiredoxin / redoxin domain-containing protein
TGME49_316330-superoxide dismutase SOD2
TGME49_316190-superoxide dismutase SOD3
1500
1000
500
0
1.5
3
6
9
12
24
36
48

Figure S14.

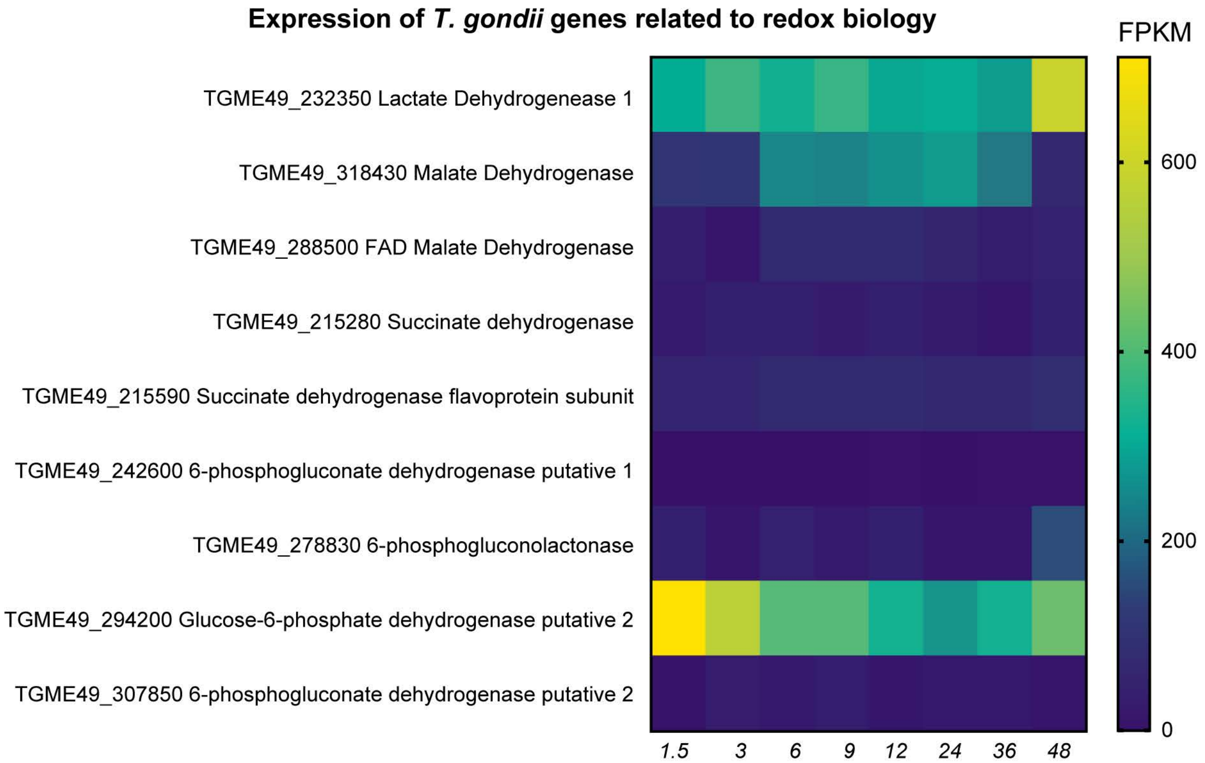
Expression of *T. gondii* genes related to redox biology
FPKM
TGME49_232350 Lactate Dehydrogenease 1
TGME49_318430 Malate Dehydrogenase
TGME49_288500 FAD Malate Dehydrogenase
TGME49_215280 Succinate dehydrogenase
TGME49_215590 Succinate dehydrogenase flavoprotein subunit
TGME49_242600 6-phosphogluconate dehydrogenase putative 1
TGME49_278830 6-phosphogluconolactonase
TGME49_294200 Glucose-6-phosphate dehydrogenase putative 2
TGME49_307850 6-phosphogluconate dehydrogenase putative 2
1.5
3
6
9
12
24
36
48
600
400
200
0

Figure S15.

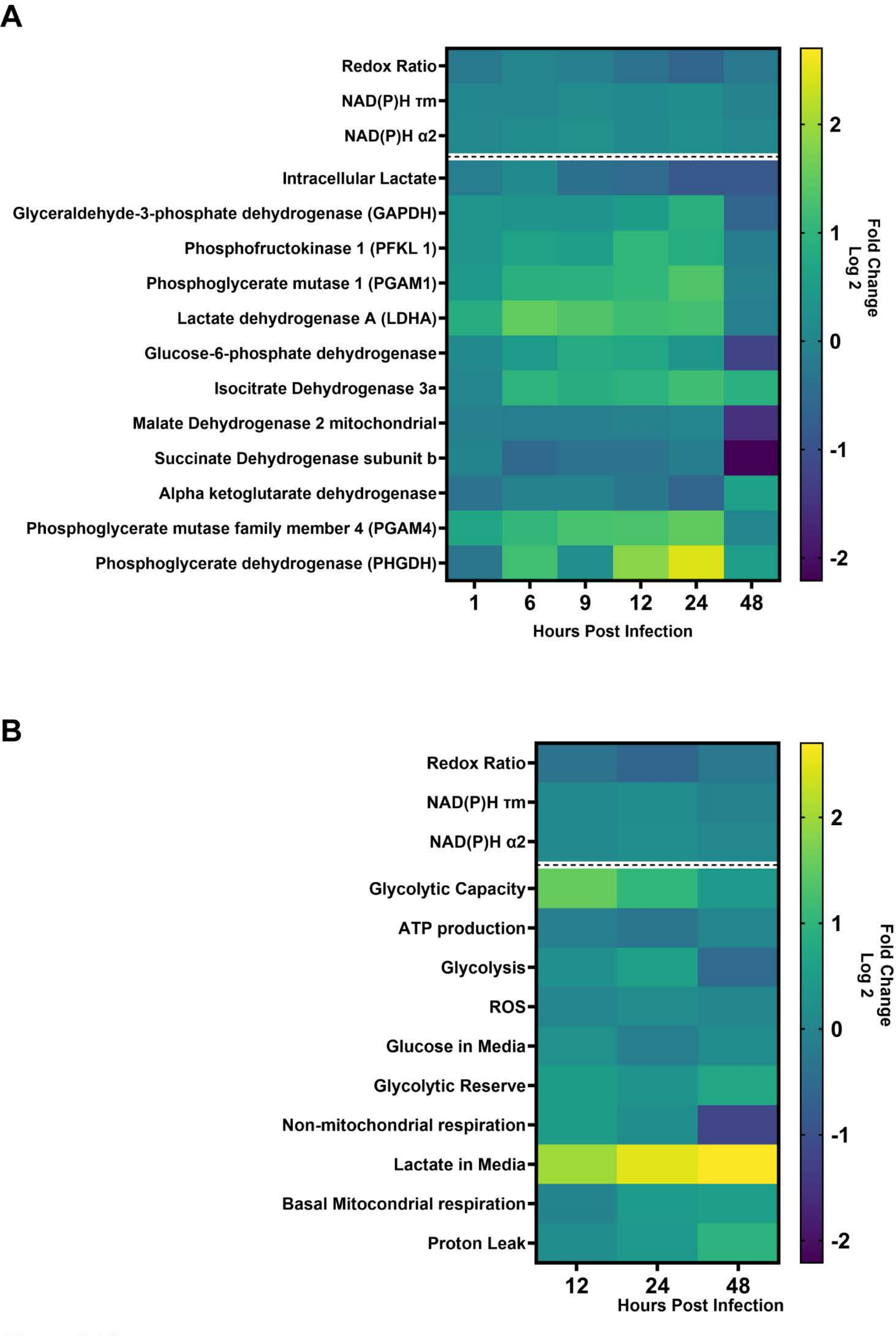
A
Redox Ratio
NAD(P)H τm
NAD(P)H α2
Intracellular Lactate
Glyceraldehyde-3-phosphate dehydrogenase (GAPDH)
Phosphofructokinase 1 (PFKL 1)
Phosphoglycerate mutase 1 (PGAM1)
Lactate dehydrogenase A (LDHA)
Glucose-6-phosphate dehydrogenase
Isocitrate Dehydrogenase 3a
Malate Dehydrogenase 2 mitochondrial
Succinate Dehydrogenase subunit b
Alpha ketoglutarate dehydrogenase
Phosphoglycerate mutase family member 4 (PGAM4)
Phosphoglycerate dehydrogenase (PHGDH)
1
6
9
12
24
48
Hours Post Infection
2
1
0
-1
-2
Fold Change
Log 2
B
Redox Ratio
NAD(P)H τm
NAD(P)H α2
Glycolytic Capacity
ATP production
Glycolysis
ROS
Glucose in Media
Glycolytic Reserve
Non-mitochondrial respiration
Lactate in Media
Basal Mitocondrial respiration
Proton Leak
12
24
48
Hours Post Infection
2
1
0
-1
-2
Fold Change
Log 2

Figure S16.

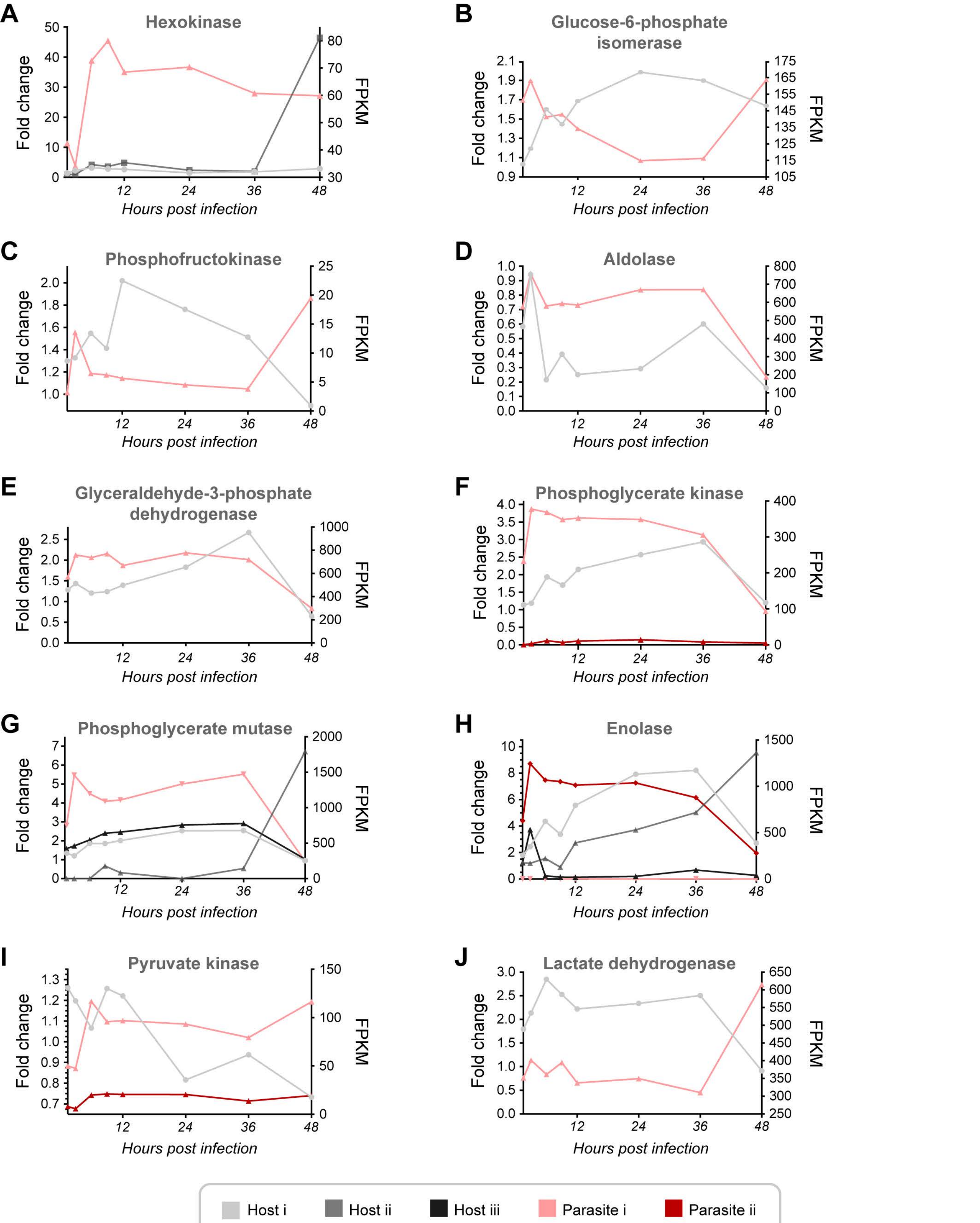
A
Hexokinase
B
Glucose-6-phosphate isomerase
C
Phosphofructokinase
D
Aldolase
E
Glyceraldehyde-3-phosphate dehydrogenase
F
Phosphoglycerate kinase
G
Phosphoglycerate mutase
H
Enolase
I
Pyruvate kinase
J
Lactate dehydrogenase
Fold change
FPKM
Hours post infection
12
24
36
48
Host i
Host ii
Host iii
Parasite i
Parasite ii

Figure S17.
