# Supplementary material for: Metabolic changes in Toxoplasma gondii-infected host cells measured by autofluorescence imaging
Source: mBio. 2024 Jul 8;15(8):e00727-24. doi: 10.1128/mbio.00727-24 (PMC11323734; doi:10.1128/mbio.00727-24)
Supplement: Supplemental Legends — Legends for the supplemental figures and the Excel spreadsheet. [file mbio.00727-24-s0002.docx]

**Supplementary Figures**

**Fig. S1. Pipeline for creating HFF whole cell and *Toxoplasma gondii* masks from two-photon FLIM images.** For each field of view three images were taken: NAD(P)H (750 ex, 440/80 em) and FAD (890 ex, 550/100 em) intensity and lifetime as well as mCherry *T. gondii* (1090 ex, 690/50 em) intensity. Scale bar = 50 µm. NAD(P)H intensity images were used to generate individual whole cell masks with semi-automated segmentation methods using CellProfiler and manual revision in Napari. *T. gondii* masks were generated from the mCherry intensity images in Python using the package scikit-image (see methods). Both masks were used to quantify the amount of *T. gondii* in each cell according to their pixel overlap.

**Fig. S2. Percentage of intracellular *Toxoplasma gondii* infection per cell during time course. (A)** The percentage of intracellular *T. gondii* per cell was calculated by dividing the sum of the pixels in the mask belonging to *T. gondii* inside the cell by the number of pixels in the whole cell mask. **(B)** The percentage of intracellular parasite pixels per area of cell in 5% bins. The histogram shows the average of two independent experiments. X-axis shows the *T. gondii* percent area per cell. The Y-axis shows the cell count. The six different timepoints (in hours) are represented by distinct colors. The pink box indicates cells with ≤5% *T. gondii* percent area per cell, which were excluded from the high *T. gondii* condition. Timepoints 1 HPI = 244 cells; timepoints 6 HPI = 225 cells; timepoints 9 HPI = 285 cells; timepoints 12 HPI = 277 cells; timepoints 24 HPI = 263 cells; timepoints 48 HPI = 312 cells.

**Fig. S3. Percentage of intracellular *Toxoplasma gondii* infection per cell during time course infection in two independent experiments.** (**A)** Experiment #1. **(B)** Experiment #2. X-axis represents the percentage of intracellular parasite by cell area by timepoints using 5% bin sizes. There are six timepoints: 1, 6, 9, 12, 24, and 48 HPI. The Y-axis represents the cell count, number of cells in each specific bin.

**Fig. S4**. **Percent of intracellular *Toxoplasma gondii* infection per cell during time course infection in experiment #1.** There are six timepoints: 1, 6, 9, 12, 24, and 48 HPI. Each timepoint is represented by one graph. X-axis represents the percentage of intracellular parasites. The Y-axis represents the cell count, number of cells in each specific percentage. **(A)** Timepoints 1 HPI = 113 cells. **(B)** Timepoints 6 HPI = 150 cells. **(C)** Timepoints 9 HPI = 187 cells. **(D)** Timepoints 12 HPI = 196 cells. **(E)** Timepoints 24 HPI = 187 cells. **(F)** Timepoints 48 HPI = 242 cells.

**Fig. S5. Percent of intracellular *Toxoplasma gondii* infection per cell during time course infection in experiment #2.** There are six timepoints: 1, 6, 9, 12, 24, and 48 HPI. Each timepoint is represented by one graph. X-axis represents the percentage of intracellular parasites. The Y-axis represents the cell count, number of cells in each specific percentage. **(A)** Timepoints 1 HPI = 131 cells. **(B)** Timepoints 6 HPI = 75 cells. **(C)** Timepoints 9 HPI = 98 cells. **(D)** Timepoints 12 HPI = 81 cells. **(E)** Timepoints 24 HPI = 76 cells. **(F)** Timepoints 48 HPI = 70 cells.

**Fig. S6. Standard analysis pipeline for calculating OMI parameters in whole cells.** NAD(P)H and FAD lifetime images were used to calculate OMI parameters by fitting the decay curves in each pixel using SPCImage. NAD(P)H and FAD parameters were then extracted from each whole cell for using masks generated in Cellprofiler and a custom Python library, *cell-analysis-tools*. Scale bar = 50 µm.

**Fig. S7. Temporal changes in ORR, NAD(P)H τ_m_, and NAD(P)H α_2_ parameters during *Toxoplasma gondii* infection using whole cell and host cell approaches. (A)**. Whole cell mask vs. host cell mask approach analysis: *T. gondii* masks were subtracted from whole cell masks to visualize changes in the host cell **(B)** Comparison of temporal ORR changes across all conditions with whole cell mask approach. **(C)**. Temporal changes in NAD(P)H τ_m_ with whole cell mask approach. **(D)** Temporal changes in NAD(P)H α_2_ with whole cell mask approach. **(E)** Comparison of temporal ORR changes across all conditions with host cell mask approach. **(F)** Temporal changes in NAD(P)H τ_m_ across all conditions with host cell mask approach. **(G)** Temporal changes in NAD(P)H α_2_ with host cell mask approach. Uninfected HFF cells are represented in blue, infected HFF cells (with *T. gondii* higher than 5%) are represented in red. Each dot represents a host cell. Total number of cells n = 243, 225, 285, 277, 263, 312 for 1, 6, 9, 12, 24 and 48 HPI, respectively. These results represent two independent experiments. Error bars represent the median and 95% confidence. Statistical significance was determined by Student’s T-test adjusted for multiple comparisons with a Bonferroni correction. *ns: p <= 1.00e+00; *: 1.00e-02 < p <= 5.00e-02; **: 1.00e-03 < p <= 1.00e-02; ***: 1.00e-04 < p <= 1.00e-03; ****: p <= 1.00e-04.* Scale bar = 50 µm.

**Fig. S8. Temporal changes in FAD lifetime of *Toxoplasma gondii*-infected HFF cells**. **A)** Representative images of FAD τ_m_ for 1 to 48 hours post infection (HPI). **(B)** FAD τ_m_ of *T. gondii*-infected HFF cells in a 48-HPI time course experiment with whole cell approach. **(C)** FAD α_1_ of *T. gondii*-infected HFF cells in a 48 HPI time course experiment whole cell approach. Uninfected HFF cells are represented in blue and infected HFF cells (with *T. gondii* higher than 5%) are represented in red. Each dot represents a host cell. These results represent two independent experiments. Scale bar = 50 µm. Error bars represent the median and 95% confidence. Statistical significance was determined by Student’s T-test adjusted for multiple comparisons with a Bonferroni correction. *ns: p <= 1.00e+00; *: 1.00e-02 < p <= 5.00e-02; **: 1.00e-03 < p <= 1.00e-02; ***: 1.00e-04 < p <= 1.00e-03; ****: p <= 1.00e-04.* N = 243, 225, 285, 277, 263, 312 for 1, 6, 9, 12, 24 and 48 HPI, respectively.

**Fig. S9. Temporal changes in ORR, NAD(P)H τ_m_, and NAD(P)H α_2_ parameters during *Toxoplasma gondii* infection and Kiss and spit using whole cell approach. (A)** Comparison of temporal ORR changes across all conditions with whole cell mask approach. **(B)**. Temporal changes in NAD(P)H τ_m_ with whole cell mask approach. **(C)** Temporal changes in NAD(P)H α_2_ with whole cell mask approach. Uninfected HFF cells are represented in blue, infected HFF cells (with *T. gondii* higher than 5%) are represented in red, Kiss and spit cells are represented in cyan color and the control of Cells + CD is represented in purple color. Each dot represents a host cell. Total number of cells n = 243, 225, 285, 277, 263, 312 for 1, 6, 9, 12, 24 and 48 HPI, respectively. These results represent two independent experiments. Error bars represent the median and 95% confidence. Statistical significance was determined by Student’s T-test adjusted for multiple comparisons with a Bonferroni correction. *ns: p <= 1.00e+00; *: 1.00e-02 < p <= 5.00e-02; **: 1.00e-03 < p <= 1.00e-02; ***: 1.00e-04 < p <= 1.00e-03; ****: p <= 1.00e-04.*

**Fig. S10. Temporal mitochondrial and glycolytic changes in HFF infected cells by different strains of *Toxoplasma gondii* during a time course of infection.** **(A)** Non-mitochondrial oxygen consumption. **(B)** Proton leak. **(C)** Glycolytic Reserve. HFF cells were infected with one of three *T. gondii* strains: ME49, RHΔMAF, or RH. Oxygen consumption rate (OCR) was calculated by Seahorse Mito Stress kit and Extracellular acidification rate (ECAR) was calculated by Seahorse Glycolysis Stress kit. Each bar represents the mean of 12 replicates and error bars represent the SEM. Statistical analysis was performed by ANOVA with Tukey’s test to compare timepoints in each strain. 0.1234 (ns), 0.0332 (*), 0.0021 (**), 0.0002 (***), <0.0001****.

**Fig. S11.** **Host GO term molecular function clustering of significant upregulated genes during ME49 *Toxoplasma gondii* infection.** Data extracted from our previous publication (20). Each of the seven timepoints are represented by a distinct color. Analysis was performed in the database DAVID.

**Fig. S12.** **Host reactome clustering of significant upregulated genes during ME49 *Toxoplasma gondii* infection.** Data extracted from our previous publication (20). Each of the seven timepoints are represented by a distinct color. Analysis was performed in the database DAVID.

**Fig. S13. Heatmap of host enzymes gene expression most likely to bind to NAD(P)H during ME49 *Toxoplasma gondii* time course infection.** Data extracted from our previous publication (20). Color scale represents the fold change with respect to the uninfected control.

**Fig. S14. Expression of 58 genes related to redox biology in *Toxoplasma gondii.*** Data extracted from our previous publication (20). The color scale represents the abundance in fragments per kilobase of exon per million mapped fragments (FPKM).

**Fig. S15**. **Expression of other genes related to redox biology in *Toxoplasma gondii.*** Data extracted from our previous publication (20). The color scale represents the abundance in fragments per kilobase of exon per million mapped fragments (FPKM).

**Fig. S16**. **Comparison of intracellular and extracellular measurements with OMI parameters in ME49 *Toxoplasma gondii* infected cells**. **(A)** Intracellular measurements and enzymes gene expression abundance were compared with ORR, NAD(P)H τ_m_, and NAD(P)H α_2_ during 48 HPI. **(B)** Extracellular measurements were compared with ORR, NAD(P)H τ_m_, and NAD(P)H α_2_ during 12, 24 and 48 HPI. Color scale represents the fold change in Log 2, with respect to the uninfected control.

**Fig. S17**. **Gene expression of both host and parasite enzymes involved in glycolysis in *Toxoplasma gondii* infection over 48-hours**. The line graphs represent mRNA abundance for the host (grey) and *T. gondii* (red). Host expression is shown as fold change (infected/uninfected) on the left Y-axis and *T. gondii* expression is shown in Fragments Per Kilobase of transcript per Million mapped reads (FPKM) values in the right Y-axis, X-axis shows hours post infection. Data extracted from our previous publication (20).

**Supplementary File 18.** Statistical Analysis of optical metabolic imaging in *T. gondii* infected HFF cells (xlsx file). Sheet 1 : Whole cell approach. Sheet 2: Host cell approach.

Excel files show the 13 OMI variables analyzed in the different conditions: all conditions, kiss and spit, media vs high *T. gondii* and Low vs High *T. gondii*. Each row indicate the comparison evaluated, the statistical method used, and the specific P value.
